# Impacts of Inflammatory Cytokines Variants on Systemic Inflammatory Profile and COVID-19 Severity

**DOI:** 10.1007/s44197-024-00204-w

**Published:** 2024-02-20

**Authors:** XueJun Deng, Kai Tang, Zhiqiang Wang, Suyu He, Zhi Luo

**Affiliations:** 1 Department of Cardiology, Suining Central Hospital, Suining, 629000 Sichuan China; 2 Orthopedic Center 1 Department of Orthopedic Trauma, Suining Central Hospital, Suining, Sichuan China; 3 The Fourth Department of Digestive Disease Center, Suining Central Hospital, Suining, 629000 Sichuan China

**Keywords:** IL-6, Cytokine storm, Coronavirus disease 2019, Severity

## Abstract

**Background:**

Cytokine storm is known to impact the prognosis of coronavirus disease 2019 (COVID-19), since pro-inflammatory cytokine variants are associated with cytokine storm. It is tempting to speculate that pro-inflammatory cytokines variants may impact COVID-19 outcomes by modulating cytokine storm. Here, we verified this hypothesis via a comprehensive analysis.

**Methods:**

PubMed, Cochrane Library, Central, CINAHL, and ClinicalTrials.gov were searched until December 15, 2023. Case–control or cohort studies that investigated the impacts of rs1800795 or rs1800629 on COVID-19 susceptibility, severity, mortality, IL-6, TNF-α, or CRP levels were included after an anonymous review by two independent reviewers and consultations of disagreement by a third independent reviewer.

**Results:**

47 studies (8305 COVID-19 individuals and 17,846 non-COVID-19 individuals) were analyzed. The rs1800629 A allele (adenine at the −308 position of the promoter was encoded by the A allele) was associated with higher levels of tumor necrosis factor-α (TNF-α) and C-reactive protein (CRP). In contrast, the rs1800795 C allele (cytosine at the −174 position of the promoter was encoded by the C allele) was linked to higher levels of interleukin-6 (IL-6) and CRP. In addition, the A allele of rs1800629 increased the severity and mortality of COVID-19. However, the C allele of rs1800795 only increased COVID-19 susceptibility.

**Conclusions:**

rs1800629 and rs1800795 variants of pro-inflammatory cytokines have significant impacts on systemic inflammatory profile and COVID-19 clinical outcomes. rs1800629 may serve as a genetic marker for severe COVID-19.

**Supplementary Information:**

The online version contains supplementary material available at 10.1007/s44197-024-00204-w.

## Introduction

The COVID-19 pandemic is caused by severe acute respiratory syndrome coronavirus 2 (SARS-CoV-2). It first appeared in December 2019 with the characteristics of a highly contagious and high mortality rate [1]. According to the World Health Organization (WHO) report, COVID-19 infected about 701 million individuals and caused more than 6.97 million deaths [2].

The clinical manifestations of COVID-19 differ substantially in severity, varying from asymptomatic or mildly symptomatic to severe or critical illness [3, 4]. Among the infected individuals with symptoms, the majority presented with mild illness [5–7], while approximately 10% progressed to a severe or critical stage requiring intensive care or mechanical ventilation support [8, 9]. The variation in symptoms or severity of COVID-19 might be attributed to some known risk factors, including males [10], older age [11], alcohol consumption [12], menopause [13], smoking [14], and underlying comorbidities (e.g., hypertension [14, 15], diabetes [14–16], cardiovascular disease [14–16], chronic pulmonary disease [15–17], obesity [15, 18], cancer [15, 19], and immunodeficiencies [15]). Although older age [11], menopause [13] and comorbidities [14–19] were associated with illness severity, these risk factors (i.e., older age, menopause and comorbidities) alone did not explain why some young [20], healthy individuals [21] suffered a severe or life-threatening illness. Interestingly, this aberrant phenomenon might be partly attributed to genetic underliers imparting inter-individual differences in susceptibility to COVID-19 infection and illness severity [22–25].

The procedure for COVID-19 infection is hierarchical. First, the spike protein S binds the angiotensin-converting enzyme 2 (ACE2) receptor to enter the host cell [26]. Then, the host immune response, such as the innate immune response, is initiated against virus infection [25]. Subsequently, some critical signaling pathways are activated, including Toll-like receptor (TLR) [24, 27–29], C-lectin type receptors (CLR) [24, 28], neuropilin-1 (NPR1) [28], and inflammasome (cytokine storm) [24, 27, 29]. Notably, the genetic variants of viral entry and innate immunity (eg, *ACE1* rs4343/rs4646994/rs1799752 [30, 31], *ACE2* rs2285666 [30], and *IFITM3* rs12252 [32]) may influence susceptibility to COVID-19 infection [25] and confer altered clinical outcomes [25].

The IL-6 gene is located on the short arm of human chromosome 7 (7p21–24), including five exons. rs1800795 (also known as -174 G > C), is located in the promoter at position -174, formed by a transversion from guanine (G) to cytosine (C) and is known to increase the transcriptional activity of IL-6 [33]. The TNF-α gene contains four exons on human chromosome 6 (6p21.31). rs1800629 (also known as -308 G > A), is located in the promoter at position -308, formed by substitution from guanine (G) to adenine (A), resulting in a 2–3 time increase in the transcriptional activity of TNF-α [34]. In addition to the rs1800795 C allele and the rs1800629 A allele may elevate plasma levels of C-reactive protein (CRP) [35, 36], it indicates that pro-inflammatory cytokines variants (i.e., 1800795 and rs1800629) may impact systemic inflammatory profile (i.e., IL-6, TNF-α, and CRP). Here, we conducted this study to investigate this hypothesis.

Cytokine storm (an aberrant systemic hyperinflammatory state characterized by high plasma levels of cytokines, including IL-1β, IL-2, IL-6, IL-7, IL-8, IL-10, IL-15, TNF-α, CRP, and MCP-1) [37–39] is closely linked to acute respiratory distress syndrome (ARDS) [40–42] and COVID-19 outcomes [43–48]. For instance, plasma levels of TNF-α, IFN-γ, IL-2, IL-4, IL-6, IL-10, and CRP were higher in COVID-19 patients compared with healthy individuals [43], indicating that cytokine storm may be related to COVID-19 infection. In contrast, plasma levels of IL-6, IL-8, IL-10, and TNF-α were higher in patients with severe COVID-19 compared with those without severe COVID-19 [44, 45], indicating that cytokine storm may be associated with COVID-19 severity. In addition, plasma levels of IL-6, IL-10, TNF-α, and CRP were higher in COVID-19 death cases than in non-death patients [46–48], suggesting that cytokine storm may be correlated with COVID-19 mortality.

IL-6 [37, 49–54] and TNF-α [37, 51, 53–55] are two critical components of cytokine storm. Since plasma levels of TNF-α [34, 56, 57] and IL-6 [33, 58, 59] are at least partly determined by variants of rs1800629 [34, 56, 57] and rs1800795 [33, 58, 59], it indicates that variants of rs1800629 and rs1800795 may impact COVID-19 outcomes by modulating cytokine storm. To verify this hypothesis, this study is required to investigate the impacts of rs1800795 and rs1800629 on systemic inflammatory profile (i.e., IL-6, TNF-α, and CRP) and COVID-19 clinical outcomes (i.e., susceptibility, severity, and mortality).

## Materials and Methods

### Study Selection

Studies that meet the following PICOS principle are preliminary selected: (1) P (population): Caucasians, Asians, Indians, and Mexicans, etc.; (2) I (intervention): no particular intervention; (3) C (comparison): the studies compare inflammatory parameters (e.g., IL-6, TNF-α, or CRP) and/or COVID-19 outcomes (e.g., susceptibility, severity, or mortality) between carriers of the rs1800795 G allele (or rs1800629 G allele) and carriers of the rs1800795 C allele (or rs1800629 A allele); (4) O (outcome): inflammatory parameters are expressed as mean with standard deviation (SD), or the number of genotype in case group, and control group is provided, to facilitate the subsequent calculation of standardized mean difference (SMD) and 95% confidence intervals (CI), or odds ratio (OR) and corresponding 95% CI; (5) S (study design): case–control studies or cohort studies, published in English, and funded by a funding body or institution.

### Literature Search

PubMed, Cochrane Library, Central, CINAHL, and ClinicalTrials.gov were searched from August 05, 2022 to December 15, 2023. The following keywords were used in the search: (“cytokines,” “inflammatory cytokines,” “pro-inflammatory cytokines”) OR (“interleukin 6,” “tumor necrosis factor-α,” “IL-6,” “TNF-α”) AND (“rs1800795,” “rs1800629,” “-174 G > C,” “-308 G > A”) AND (“variant,” “variation,” “mutant,” “mutation,” “polymorphism,” “SNP”) OR (“single nucleotide polymorphism”) AND (“IL-6,” “TNF-α,” “CRP,” “interleukin 6,” “tumour necrosis factor-α,” “C-reactive protein”) OR (“COVID-19,” “SAR-CoV-2,” “coronavirus disease 2019”) OR (“severe acute respiratory syndrome coronavirus 2”) AND/OR (“clinical outcomes,” “susceptibility,” “severity,” “mortality”).

### Inclusion and Exclusion Criteria

The inclusion criteria for the impacts of inflammatory cytokines variants on inflammatory biomarkers include:

1. Article type: case–control studies or cohort studies that investigated the effects of rs1800629 or rs1800795 on IL-6, TNF-α, or CRP levels.
2. Data type: studies that provided mean IL-6, TNF-α, or CRP levels with SD.
3. Inflammatory biomarkers: studies that at least provided two of three parameters in the inflammatory profiles (i.e., IL-6, TNF-α, and CRP).
4. Genetic information: studies that provided the genotype frequencies of rs1800795 and rs1800629.
5. Human subjects: test subjects were limited to humans.
6. Language: the language of eligible studies was restricted to English.

The inclusion criteria for the impacts of inflammatory cytokines variants on COVID-19 clinical outcomes include:

1. Article type: case–control studies that investigated the effects of rs1800795 or rs1800629 on COVID-19 susceptibility, severity, or mortality.
2. Population: COVID-19 patients were confirmed by RT-PCR or PCR–RFLP.
3. Genetic information: studies that provided case and control genotype frequencies.
4. Language: studies published in English language only.

Studies were rejected if they met one or more of the following exclusion criteria:

1. Studies did not relate to rs1800795 or rs1800629.
2. Studies did not relate to inflammatory biomarkers or COVID-19.
3. Studies did not present genetic information.
4. Studies with incomplete data.
5. Pedigree or overlapping studies.
6. Abstract/comments/review/case report/animal studies.

### Data Extraction

Two authors (XD and KT) independently extracted the data using standardized data extraction sheets (Table S1–S11). The discrepancy in data collected was resolved by consensus or a discussion with the senior author (ZL). The following data were extracted from each eligible study: the first author’s name (Table S1), year (Table S1), country (Table S1), ethnicity (Table S1), gender (Table S1), genotyping methods (Table S1), total sample size (Table S1), mean inflammatory biomarkers levels with SD or SE by genotype (Table S2–S5), and genotype counts (Table S6–S11).

### Data Analysis

The SMD and 95% CI were used to evaluate the differences in inflammatory biomarkers between different genotypes. The OR with 95% CI was used to evaluate the impacts of rs1800795 and rs1800629 on COVID-19 susceptibility, severity, and mortality. The pooled OR was performed for the allelic model [(*A vs. G*) for rs1800629, (*C vs. G*) for rs1800795], additive model [(*AA vs. GG*) for rs1800629, (*CC vs. GG*) for rs1800795], dominant model [(*GA* + *AA*) vs. *GG* for rs1800629, (*GC* + *CC*) vs. *GG* for rs1800795] and recessive model [(*GG* + *GA*) vs. *AA* for rs1800629, (*GG* + *GC*) vs. *CC* for rs1800795]. Since most of the included studies presented inflammatory biomarkers in a dominant model [(*GA* + *AA*) vs. *GG* for rs1800629, (*GC* + *CC*) vs. *GG* for rs1800795], a dominant model was adopted to ensure adequate statistical power. All statistical tests were conducted with the Cochrane Collaboration meta-analysis software, Review Manager 5.4. *P* < 0.05 was recognized as statistically significant.

### Subgroup Analysis

Subgroup analysis was carried out on Caucasians, Asians, Indians, and Mexicans. In some studies, the subjects were divided into more than one subpopulation (e.g., the subjects originated from different ethnicities or genders). Each subpopulation was regarded as an independent comparison in this study.

### Heterogeneity Processing

Heterogeneity was tested by *I*^2^ statistic and Cochran's χ^2^-based Q statistic. If heterogeneity was significant (*I*^2^ > 50%, *P* ≤ 0.05), the random-effect model (DerSimonian-Laird method) was used to calculate the results. Otherwise, the fixed-effect model (Mantel–Haenszel method) would be adopted (*I*^2^ < 50%, *P* > 0.05) [60]. In addition, the Galbraith plot was employed to detect the potential sources of heterogeneity. To completely eliminate the impact of heterogeneity on the results, all results were recalculated after excluding the studies with heterogeneity.

### Publication Bias Test

The Begg funnel plot and Egger linear test evaluated the probability of publication bias among the included studies [61].

### Risk of Bias/Quality Assessment

The risk bias among the included studies was evaluated by the risk-of-bias plot [62], in which different colors represent different levels of risk bias. For instance, green indicates a low risk bias, while red suggests a high risk bias.

## Results

### Study Selection

**Fig. 1:**
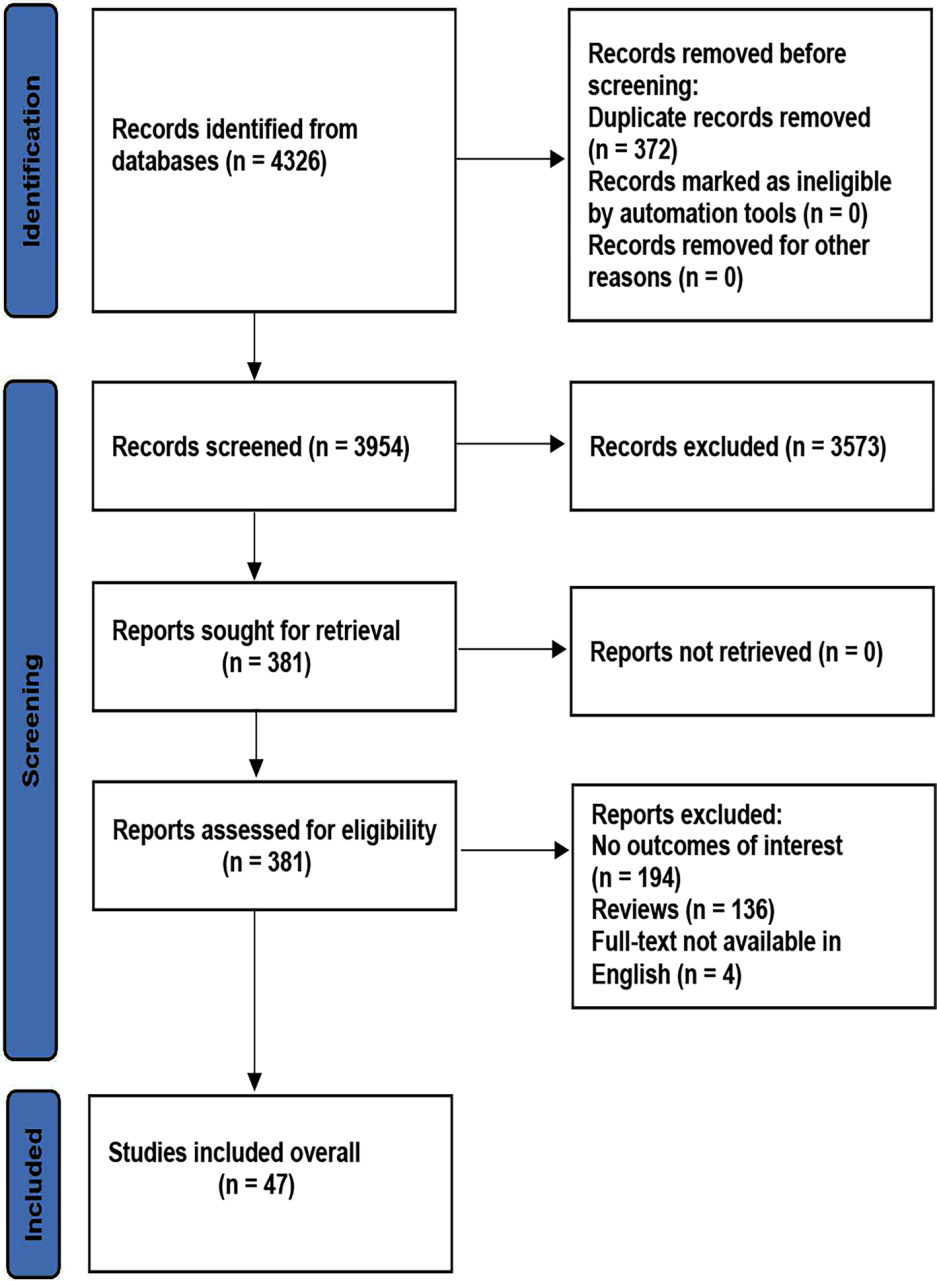
Flow diagram of the literature search process

Preferred Reporting Items for Systematic Reviews and Meta-analyses (PRISMA) is an essential reference material and reporting standard for conducting meta-analysis, including seven parts and 27 projects. The present meta-analysis follows the PRISMA Checklist 2020 (Table S12). The initial search of the databases yielded 4326 studies. After screening, 3573 studies were excluded by their title, abstract, and content. The remaining 381 studies were re-estimated by the inclusion criteria. Three hundred and thirty-four studies were further excluded due to the following reasons: 194 studies did not provide outcomes of interest, 136 studies were reviews, and four studies did not provide full-text in English. Finally, 47 studies (26,151 individuals) were included in this study (Fig. 1).

### Characteristics of the Included Studies

The characteristics of the included studies are presented in Supplementary Material: Table S1. The plasma TNF-α levels by the genotype of rs1800629 are presented in Supplementary Material: Table S2. The plasma CRP levels by the genotype of rs1800629 are presented in Supplementary Material: Table S3. The plasma IL-6 levels by the genotype of rs1800795 are presented in Supplementary Material: Table S4. The plasma CRP levels by the genotype of rs1800795 are presented in Supplementary Material: Table S5. The genotype distribution frequency of rs1800629 in COVID-19 and non-COVID-19 individuals is presented in Supplementary Material: Table S6. The genotype distribution frequency of rs1800795 in COVID-19 and non-COVID-19 individuals is presented in Supplementary Material: Table S7. The genotype distribution frequency of rs1800629 in severe and non-severe COVID-19 individuals is presented in Supplementary Material: Table S8. The genotype distribution frequency of rs1800795 in severe and non-severe COVID-19 individuals is presented in Supplementary Material: Table S9. The genotype distribution frequency of rs1800629 in COVID-19 dead and non-dead individuals is presented in Supplementary Material: Table S10. The genotype distribution frequency of rs1800795 in COVID-19 dead and non-dead individuals is presented in Supplementary Material: Table S11. The PRISMA Checklist 2020 is presented in Supplementary Material: Table S12. The forest plot of the meta-analysis between rs1800629 and COVID-19 susceptibility is presented in Supplementary Material: Figure S1. The forest plot of the meta-analysis between rs1800795 and COVID-19 severity is presented in Supplementary Material: Figure S2. The forest of the meta-analysis between rs1800795 and COVID-19 mortality is presented in Supplementary Material: Figure S3. The risk bias plot of the meta-analysis between rs1800795 and interleukin-6 levels is presented in Supplementary Material: Figure S4. The risk bias plot of the meta-analysis between rs1800795 and C-reactive protein levels is presented in Supplementary Material: Figure S5. The risk bias plot of the meta-analysis between rs1800629 with COVID-19 severity is presented in Supplementary Material: Figure S6. The risk bias plot of the meta-analysis between rs1800629 with tumor necrosis factor-αis presented in Supplementary Material: Figure S7. The risk bias plot of the meta-analysis between rs1800629 with C-reactive protein levels is presented in Supplementary Material: Figure S8. The risk bias plot of the meta-analysis between rs1800795 with COVID-19 severity is presented in Supplementary Material: Figure S9.

### Impacts of rs1800629 and rs1800795 on Inflammatory Biomarkers

**Fig. 2:**
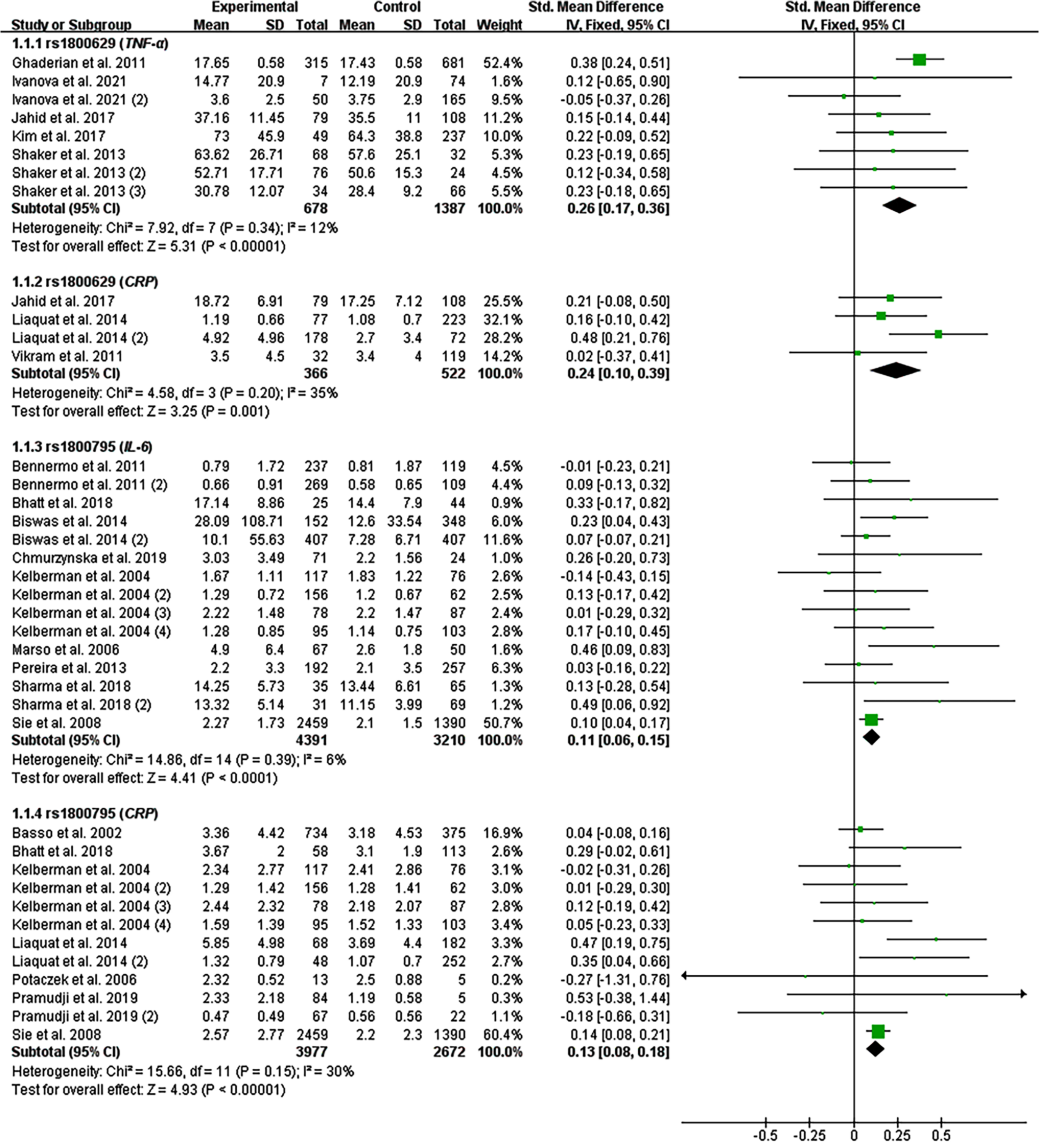
Forest plot of the meta-analysis between inflammatory cytokines variants and inflammatory biomarkers

All the results stated below were the data excluded heterogeneity. The consistent finding for the impacts of rs1800629 (Table 1, Fig. 2) and rs1800795 (Table 2, Fig. 2) on inflammatory biomarkers was increased CRP levels. In addition, the rs1800629 A allele (Table 1, Fig. 2) and rs1800795 C allele (Table 2, Fig. 2) elevated TNF-α and IL-6 levels, respectively. Subgroup analysis indicated that the impacts of rs1800629 (Table 1) and rs1800795 (Table 2) on inflammatory biomarkers were significant in Caucasians.

**Table 1:** Impacts of TNF-α rs1800629 variant on systemic inflammatory profiles and COVID-19 clinical outcomes

Groups or subgroups	*P*_H_	OR (95% CI)	*P*_OR_	Groups or subgroups	*P*_H_	OR (95% CI)	*P*_OR_
Overall results				Recalculated results			
TNF-α				TNF-α			
Dominant model (*GA* + *AA vs. GG*)				Dominant model (*GA* + *AA vs. GG*)			
All	< 0.001	0.62 (0.27–0.97)	< 0.01	All	0.34	0.26 (0.17–0.36)	< 0.001
Caucasian	< 0.001	0.44 (0.08–0.81)	0.02	Caucasian	0.22	0.29 (0.18–0.40)	< 0.001
Indian	< 0.001	0.41 (−0.53–1.34)	0.39	Indian	–	–	–
CRP				CRP			
Dominant model (*GA* + *AA vs. GG*)				Dominant model (*GA* + *AA vs. GG*)			
All	< 0.01	0.39 (0.17–0.60)	< 0.001	All	0.20	0.24 (0.10–0.39)	< 0.01
Caucasian	0.01	0.44 (0.15–0.73)	< 0.01	Caucasian	0.09	0.31 (0.12–0.50)	< 0.01
Indian	0.02	0.31 (−0.06–0.69)	0.10	Indian	0.46	0.14 (−0.09–0.38)	0.23
COVID-19 susceptibility				COVID-19 susceptibility			
Allelic model (*A vs. G*)				Allelic model (*A vs. G*)			
All	< 0.001	1.19 (0.81–1.75)	0.38	All	0.06	0.85 (0.67–1.07)	0.16
Caucasian	< 0.001	1.25 (0.76–2.06)	0.39	Caucasian	0.77	1.59 (1.07–2.37)	0.02
Asian	0.04	1.02 (0.46–2.25)	0.97	Asian	0.04	0.97 (0.72–1.31)	0.84
Additive model (*AA vs. GG*)				Additive model (*AA vs. GG*)			
All	< 0.001	1.04 (0.41–2.64)	0.93	All	0.33	1.01 (0.59–1.73)	0.97
Caucasian	< 0.001	1.07 (0.36–3.19)	0.91	Caucasian	0.33	1.06 (0.57–1.95)	0.86
Asian	0.16	0.96 (0.13–7.2)	0.97	Asian	0.16	0.79 (0.25–2.46)	0.68
Heterozygote model (*GA vs. GG*)				Heterozygote model (*GA vs. GG*)			
All	< 0.001	1.35 (0.89–2.06)	0.16	All	0.15	0.78 (0.60–1.02)	0.07
Caucasian	< 0.001	1.42 (0.78–2.59)	0.25	Caucasian	0.38	1.88 (1.20–2.97)	0.01
Asian	0.36	1.01 (0.72–1.43)	0.94	Asian	0.36	1.01 (0.72–1.40)	0.97
Recessive model (*AA vs. GG* + *GA*)				Recessive model (*AA vs. GG* + *GA*)			
All	< 0.01	0.98 (0.50–1.92)	0.96	All	0.30	1.01 (0.59–1.73)	0.97
Caucasian	< 0.001	0.98 (0.46–2.07)	0.96	Caucasian	0.27	1.06 (0.58–1.94)	0.86
Asian	0.17	0.96 (0.13–7.00)	0.96	Asian	0.17	0.78 (0.25–2.45)	0.67
Dominant model (*GA* + *AA vs. GG*)				Dominant model (*GA* + *AA vs. GG*)			
All	< 0.001	1.30 (0.82–2.05)	0.26	All	0.11	0.80 (0.62–1.04)	0.09
Caucasian	< 0.001	1.37 (0.73–2.60)	0.33	Caucasian	0.74	1.80 (1.16–2.79)	0.01
Asian	0.16	1.06 (0.57–1.97)	0.86	Asian	0.16	0.99 (0.72–1.36)	0.94
Overdominant model (*GA vs. GG* + *AA*)				Overdominant model (*GA vs. GG* + *AA*)			
All	0.01	1.23 (0.93–1.64)	0.15	All	0.36	1.08 (0.93–1.26)	0.33
Asian	0.37	1.01 (0.73–1.41)	0.94	Asian	0.37	1.01 (0.73–1.41)	0.94
Caucasian	< 0.01	1.28 (0.87–1.89)	0.20	Caucasian	0.18	0.90 (0.75–1.07)	0.22
COVID-19 severity				COVID-19 severity			
Allelic model (*A vs. G*)				Allelic model (*A vs. G*)			
All	< 0.001	1.71 (0.72–4.06)	0.22	All	0.34	1.21 (0.99–1.48)	0.06
Caucasian	< 0.001	1.66 (0.54–5.05)	0.37	Caucasian	0.11	1.16 (0.92–1.45)	0.21
Asian	0.67	1.45 (0.39–5.40)	0.58	Asian	0.67	1.47 (0.40–5.39)	0.56
Mexican	0.40	1.39 (0.86–2.23)	0.18	Mexican	0.40	1.41 (0.88–2.27)	0.15
Additive model (*AA vs. GG*)				Additive model (*AA vs. GG*)			
All	< 0.001	2.44 (0.21–28.16)	0.47	All	0.93	1.09 (0.58–2.05)	0.79
Caucasian	< 0.001	3.56 (0.17–74.49)	0.41	Caucasian	0.81	1.14 (0.59–2.20)	0.71
Heterozygote model (*GA vs. GG*)				Heterozygote model (*GA vs. GG*)			
All	< 0.001	1.60 (0.89–2.89)	0.12	All	0.25	1.26 (0.99–1.59)	0.06
Caucasian	< 0.001	1.63 (0.69–3.86)	0.27	Caucasian	0.09	1.18 (0.89–1.55)	0.25
Asian	0.98	2.73 (0.57–13.15)	0.21	Asian	0.98	2.73 (0.57–13.17)	0.21
Mexican	0.37	137 (0.84–2.22)	0.21	Mexican	0.37	1.40 (0.86–2.26)	0.18
Recessive model (*AA vs. GG* + *GA*)				Recessive model (*AA vs. GG* + *GA*)			
All	< 0.001	1.66 (0.45–6.10)	0.45	All	0.94	0.84 (0.46–1.54)	0.58
Caucasian	< 0.001	2.01 (0.48–8.49)	0.34	Caucasian	0.86	1.25 (0.67–2.33)	0.49
Dominant model (*GA* + *AA vs. GG*)				Dominant model (*GA* + *AA vs. GG*)			
All	< 0.001	1.83 (0.86–3.93)	0.12	All	0.27	1.26 (1.00–1.58)	0.05
Caucasian	< 0.001	2.01 (0.64–6.29)	0.23	Caucasian	0.08	1.19 (0.91–1.55)	0.21
Asian	0.81	1.97 (0.47–8.30)	0.36	Asian	0.81	1.99 (0.47–8.32)	0.35
Mexican	0.37	1.39 (0.85–2.25)	0.19	Mexican	0.37	1.41 (0.87–2.29)	0.16
Overdominant model (*GA vs. GG* + *AA*)				Overdominant model (*GA vs. GG* + *AA*)			
All	< 0.01	1.10 (0.75–1.60)	0.62	All	0.21	0.81 (0.65–1.02)	0.08
Caucasian	< 0.01	0.98 (0.63–1.52)	0.91	Caucasian	0.08	1.15 (0.88–1.50)	0.32
Asian	0.99	2.81 (0.58–13.51)	0.20	Asian	0.99	2.81 (0.58–13.53)	0.20
Mexican	0.37	1.37 (0.84–2.22)	0.21	Mexican	0.37	1.40 (0.86–2.26)	0.18
COVID-19 mortality				COVID-19 mortality			
Allelic model (*A vs. G*)				Allelic model (*A vs. G*)			
All	< 0.001	2.64 (0.54–12.84)	0.23	All	0.76	1.07 (0.70–1.65)	0.74
Caucasian	< 0.001	4.43 (0.10–190.76)	0.44	Caucasian	0.96	0.95 (0.54–1.65)	0.84
Asian	0.44	133 (0.69–2.56)	0.40	Asian	0.44	1.30 (0.68–2.51)	0.43
Additive model (*AA vs. GG*)				Additive model (*AA vs. GG*)			
All	< 0.001	2.35 (0.15–36.72)	0.54	All	0.94	0.71 (0.20–2.57)	0.60
Caucasian	< 0.01	3.05 (0.08–122.11)	0.55	Caucasian	0.88	0.65 (0.16–2.70)	0.56
Heterozygote model (*GA vs. GG*)				Heterozygote model (*GA vs. GG*)			
All	0.92	1.44 (0.85–2.43)	0.18	All	0.92	1.44 (0.85–2.43)	0.18
Caucasian	0.78	1.38 (0.62–3.07)	0.43	Caucasian	0.78	1.38 (0.62–3.07)	0.43
Asian	0.53	1.48 (0.74–2.98)	0.27	Asian	0.53	1.48 (0.74–2.98)	0.27
Recessive model (*AA vs. GG* + *GA*)				Recessive model (*AA vs. GG* + *GA*)			
All	< 0.001	2.72 (0.09–80.55)	0.56	All	0.92	1.65 (0.49–5.61)	0.42
Caucasian	< 0.001	3.77 (0.04–351.71)	0.57	Caucasian	0.86	0.56 (0.15–2.13)	0.39
Dominant model (*GA* + *AA vs. GG*)				Dominant model (*GA* + *AA vs. GG*)			
All	0.07	2.30 (1.45–3.66)	< 0.001	All	0.07	2.30 (1.45–3.66)	< 0.001
Caucasian	0.01	3.17 (1.62–6.18)	< 0.01	Caucasian	0.01	3.17 (1.62–6.18)	< 0.01
Asian	0.51	1.41 (0.70–2.83)	0.33	Asian	0.51	1.41 (0.70–2.83)	0.33
Overdominant model (*GA vs. GG* + *AA*)				Overdominant model (*GA vs. GG* + *AA*)			
All	< 0.001	0.92 (0.21–3.97)	0.91	All	0.92	0.66 (0.40–1.11)	0.11
Caucasian	< 0.001	0.43 (0.02–10.55)	0.60	Caucasian	0.77	1.53 (0.72–3.26)	0.27
Asian	0.53	1.51 (0.75–3.04)	0.25	Asian	0.53	1.49 (0.74–3.00)	0.26

*TNF-α* tumor necrosis factor-α gene; *CRP* C-reactive protein; *P*_*H*_ P for heterogeneity; *OR* odds ratio

**Table 2:** Impacts of IL-6 rs1800795 variant on systemic inflammatory profiles and COVID-19 clinical outcomes

Groups or subgroups	*P*_H_	OR (95% CI)	*P*_OR_	Groups or subgroups	*P*_H_	OR (95% CI)	*P*_OR_
Overall results				Recalculated results			
IL-6				IL-6			
Dominant model (*GC* + *CC vs. GG*)				Dominant model (*GC* + *CC vs. GG*)			
All	< 0.001	0.24 (0.14–0.35)	< 0.001	All	0.39	0.11 (0.06–0.15)	< 0.001
Caucasian	< 0.001	0.20 (0.06–0.33)	< 0.01	Caucasian	0.40	0.10 (0.04–0.15)	< 0.01
Indian	< 0.001	0.37 (0.15–0.60)	< 0.01	Indian	0.29	0.16 (0.15–0.26)	< 0.01
CRP				CRP			
Dominant model (*GC* + *CC vs. GG*)				Dominant model (*GC* + *CC vs. GG*)			
All	< 0.001	0.23 (0.08–0.37)	< 0.01	All	0.15	0.13 (0.08–0.18)	< 0.01
Caucasian	< 0.001	0.20 (0.04–0.36)	< 0.01	Caucasian	0.13	0.13 (0.04–0.22)	0.01
Indian	0.06	0.55 (−0.01–1.11)	0.06	Indian	–	–	–
COVID-19 susceptibility				COVID-19 susceptibility			
Allelic model (*C vs. G*)				Allelic model (*C vs. G*)			
All	< 0.001	0.85 (0.24–2.97)	0.80	All	0.74	1.63 (1.22–2.19)	< 0.01
Additive model (*CC vs. GG*)				Additive model (*CC vs. GG*)			
All	0.01	0.72 (0.06–8.58)	0.80	All	0.69	1.72 (1.31–2.27)	< 0.001
Heterozygote model (GC vs. GG)				Heterozygote model (*GC vs. GG*)			
All	< 0.001	0.78 (0.21–2.85)	0.71	All	0.21	2.72 (1.76–4.22)	< 0.001
Recessive model (*CC vs. GG* + *GC*)				Recessive model (*CC vs. GG* + *GC*)			
All	0.06	0.88 (0.13–5.85)	0.90	All	0.70	0.44 (0.19–1.03)	0.06
Dominant model (*GC* + *CC vs. GG*)				Dominant model (*GC* + *CC vs. GG*)			
All	< 0.001	0.78 (0.19–3.26)	0.73	All	0.63	1.71 (1.21–2.41)	< 0.01
Overdominant model (*GC vs. GG* + *CC*)				Overdominant model (*GC vs. GG* + *CC*)			
All	0.01	0.85 (0.30–2.44)	0.76	All	0.32	2.23 (1.47–3.37)	< 0.001
COVID-19 severity				COVID-19 severity			
Allelic model (*C vs. G*)				Allelic model (*C vs. G*)			
All	0.01	0.96 (0.64–1.45)	0.85	All	0.11	0.86 (0.68–1.09)	0.22
Caucasian	0.18	1.01 (0.62–1.65)	0.97	Caucasian	0.18	1.01 (0.74–1.36)	0.96
Additive model (*CC vs. GG*)				Additive model (*CC vs. GG*)			
All	0.08	0.83 (0.45–1.52)	0.54	All	0.08	0.83 (0.45–1.51)	0.54
Caucasian	0.05	0.99 (0.49–2.02)	0.98	Caucasian	0.05	0.99 (0.49–2.02)	0.98
Heterozygote model (*GC vs. GG*)				Heterozygote model (*GC vs. GG*)			
All	0.01	0.92 (0.53–1.59)	0.76	All	0.09	0.83 (0.61–1.13)	0.24
Caucasian	0.90	1.01 (0.68–1.49)	0.98	Caucasian	0.90	1.01 (0.68–1.49)	0.98
Recessive model (*CC vs. GG* + *GC*)				Recessive model (*CC vs. GG* + *GC*)			
All	0.08	0.83 (0.46–1.51)	0.55	All	0.08	1.20 (0.66–2.18)	0.55
Caucasian	0.05	0.99 (0.49–1.98)	0.97	Caucasian	0.05	0.99 (0.49–1.98)	0.97
Dominant model (*GC* + *CC vs. GG*)				Dominant model (*GC* + *CC vs. GG*)			
All	< 0.01	0.90 (0.52–1.56)	0.72	All	0.06	0.83 (0.62–1.11)	0.20
Caucasian	0.55	1.01 (0.70–1.45)	0.98	Caucasian	0.55	1.01 (0.70–1.46)	0.97
Overdominant model (*GC vs. GG* + *CC*)				Overdominant model (*GC vs. GG* + *CC*)			
All	0.01	0.93 (0.54–1.59)	0.79	All	0.09	1.18 (0.87–1.59)	0.29
Caucasian	0.93	1.00 (0.68–1.47)	0.99	Caucasian	0.93	1.00 (0.68–1.47)	0.99
COVID-19 mortality				COVID-19 mortality			
Allelic model (*C vs. G*)				Allelic model (*C vs. G*)			
All	0.08	1.01 (0.52–1.96)	0.98	All	0.08	1.01 (0.52–1.96)	0.98
Caucasian	0.08	1.01 (0.52–1.96)	0.98	Caucasian	0.08	1.01 (0.52–1.96)	0.98
Additive model (*CC vs. GG*)				Additive model (*CC vs. GG*)			
All	0.25	1.09 (0.29–4.10)	0.90	All	0.25	1.09 (0.29–4.10)	0.90
Caucasian	0.25	1.09 (0.29–4.10)	0.90	Caucasian	0.25	1.09 (0.29–4.10)	0.90
Heterozygote model (*GC vs. GG*)				Heterozygote model (*GC vs. GG*)			
All	0.21	0.90 (0.35–2.31)	0.83	All	0.21	0.90 (0.35–2.31)	0.83
Caucasian	0.21	0.90 (0.35–2.31)	0.83	Caucasian	0.21	0.90 (0.35–2.31)	0.83
Recessive model (*CC vs. GG* + *GC*)				Recessive model (*CC vs. GG* + *GC*)			
All	0.53	0.86 (0.26–2.84)	0.81	All	0.53	0.86 (0.26–2.84)	0.81
Caucasian	0.53	0.86 (0.26–2.84)	0.81	Caucasian	0.53	0.86 (0.26–2.84)	0.81
Dominant model (*GC* + *CC vs. GG*)				Dominant model (*GC* + *CC vs. GG*)			
All	0.12	0.96 (0.41–2.28)	0.93	All	0.12	0.96 (0.41–2.28)	0.93
Caucasian	0.12	0.96 (0.41–2.28)	0.93	Caucasian	0.12	0.96 (0.41–2.28)	0.93
Overdominant model (*GC vs. GG* + *CC*)				Overdominant model (*GC vs. GG* + *CC*)			
All	0.42	1.10 (0.46–2.66)	0.83	All	0.42	1.10 (0.46–2.66)	0.83
Caucasian	0.42	1.10 (0.46–2.66)	0.83	Caucasian	0.42	1.10 (0.46–2.66)	0.83

*IL-6* interleukin-6 gene; *CRP* C-reactive protein; *P*_*H*_ P for heterogeneity; *OR* odds ratio

### Impacts of rs1800629 and rs1800795 on COVID-19 Susceptibility

**Fig. 3:**
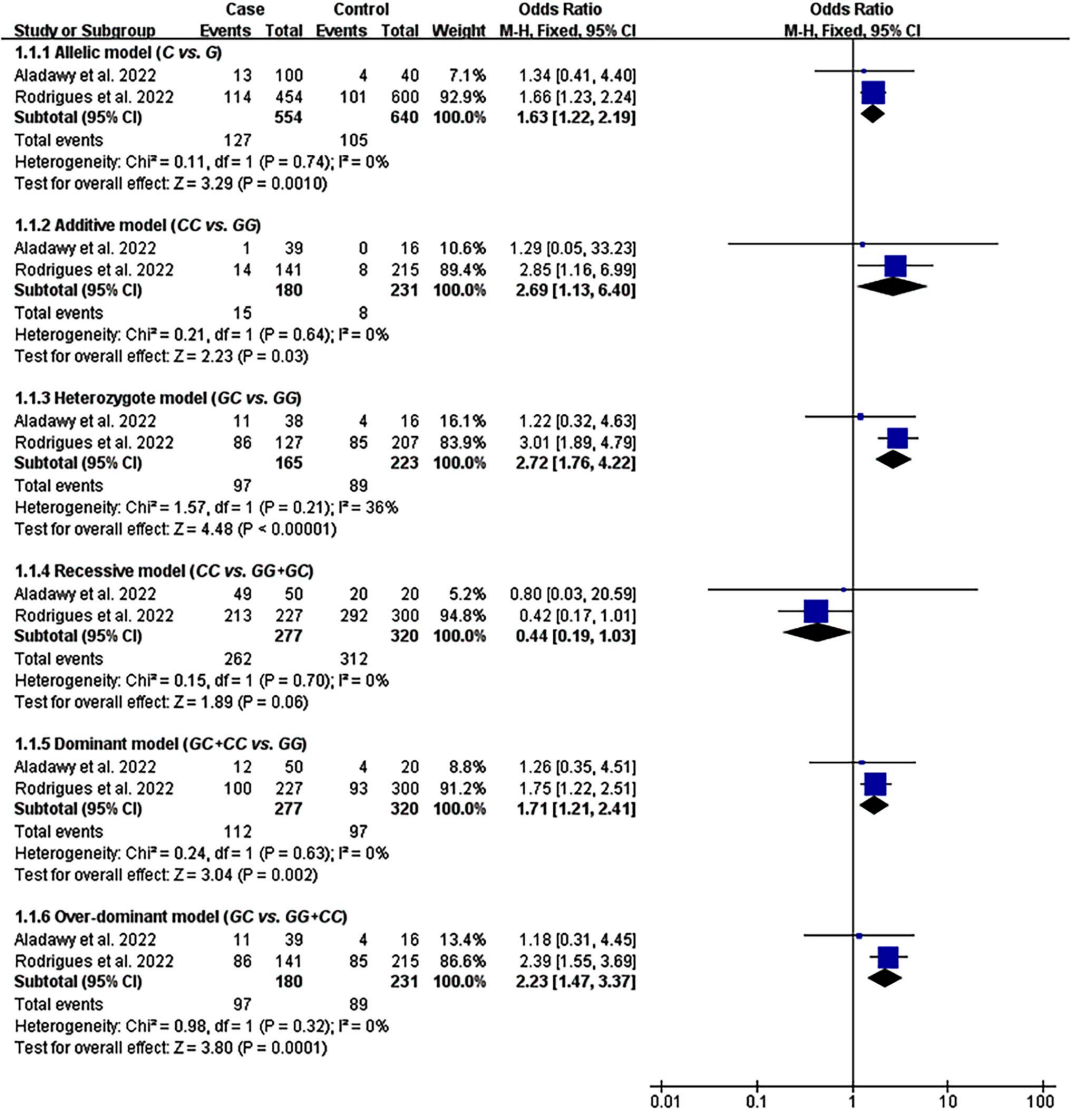
Forest plot of the meta-analysis between rs1800795 and COVID-19 susceptibility

The impact of rs1800795 on COVID-19 susceptibility was significant in five genetic models (Table 2, Fig. 3). However, the impact of rs1800629 on COVID-19 susceptibility did not show statistically significant in all genetic models (Table 1, Figure S1). Subgroup analysis indicated that the A allele of rs1800629 significantly increased COVID-19 risk in Caucasians under the allelic, heterozygote, and dominant models (Table 1).

### Impacts of rs1800629 and rs1800795 on COVID-19 Severity

**Fig. 4:**
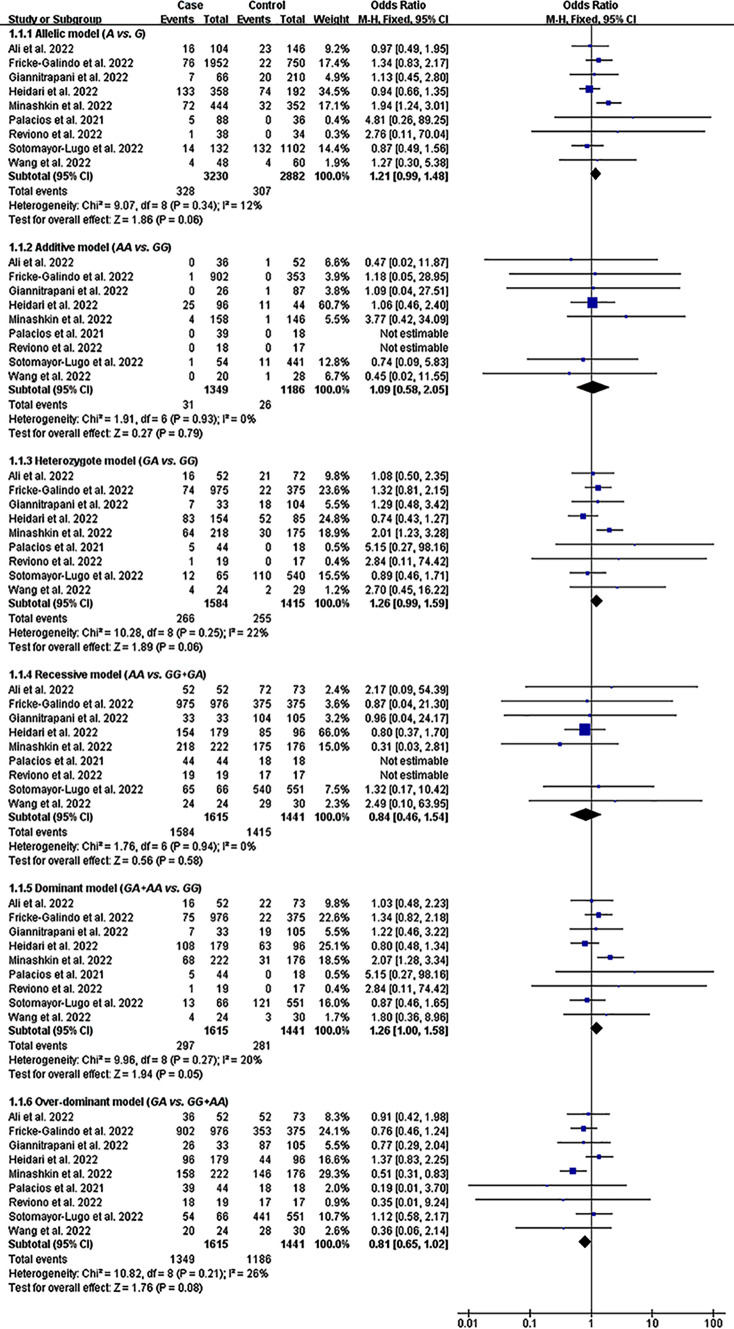
Forest plot of the meta-analysis between rs1800629 and COVID-19 severity

The A allele of rs1800629 significantly increased the severity of COVID-19 under the dominant model (Table 1, Fig. 4). In addition, a marginally significant impact was detected between rs1800629 and COVID-19 severity under the allelic, heterozygote, and over-dominant models (Table 1, Fig. 4). In contrast, rs1800795 did not show a statistically significant impact on COVID-19 severity (Table 2, Figure S2).

### Impacts of rs1800629 and rs1800795 on COVID-2019 Mortality

**Fig. 5:**
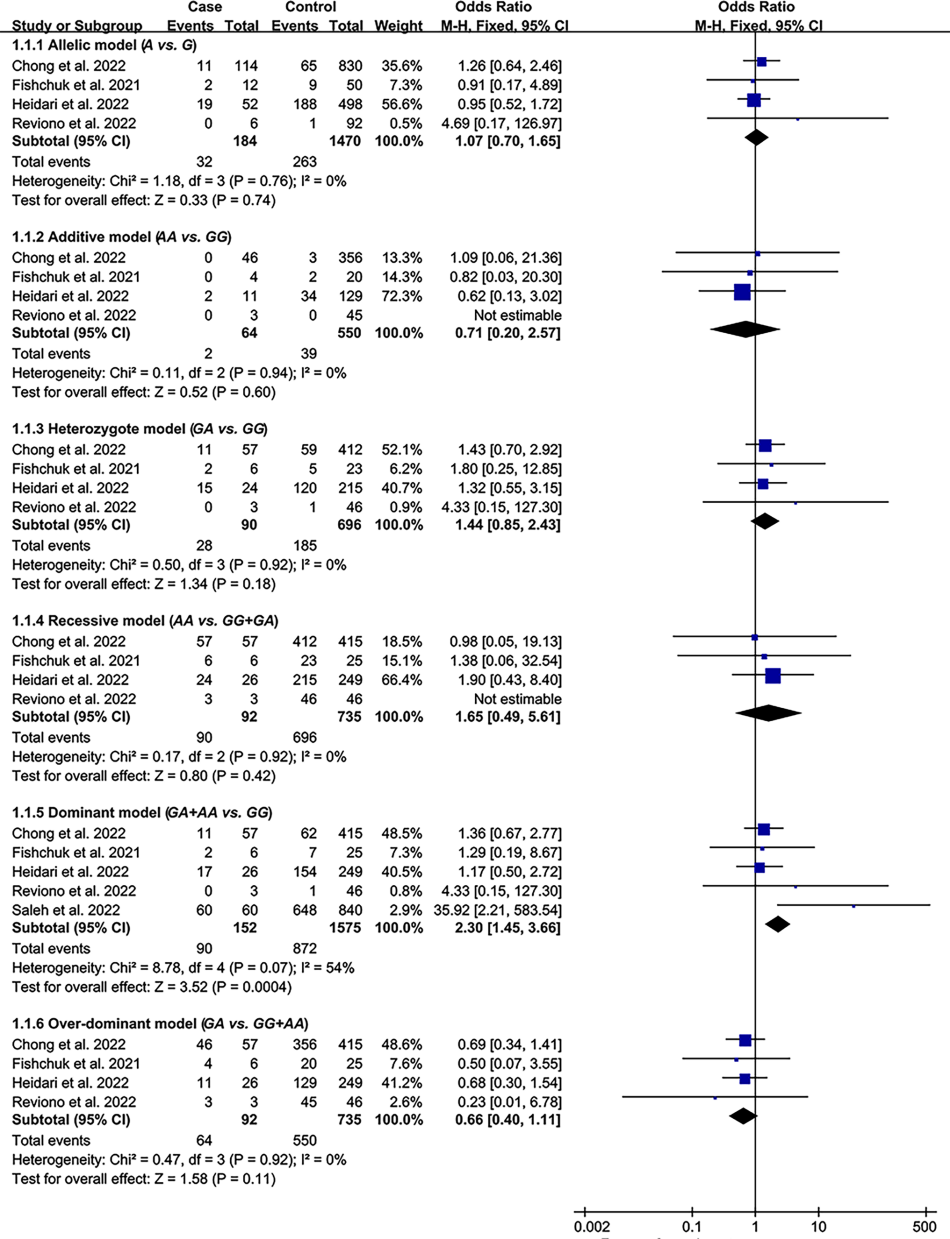
Forest plot of the meta-analysis between rs1800629 and COVID-19 mortality

The rs1800629 A allele significantly increased the mortality of COVID-19 under the dominant model (Table 1, Fig. 5). Subgroup analysis indicated that the impact of rs1800629 on COVID-19 mortality was significant in Caucasians (Table 1). However, rs1800795 did not show a statistically significant impact on COVID-19 mortality (Table 2, Figure S3).

### Evaluation of Heterogeneity

In analyzing the impacts of rs1800629 on COVID-19 clinical outcomes, two [67] and Heidari et al. (2022), one [67], and one [67] comparison were identified as the main heterogeneity contributor to COVID-19 susceptibility, severity, and mortality. Notably, the recalculated results for susceptibility and severity changed substantially after excluding those comparisons (please see Table 1 for more details).

In analyzing the impacts of rs1800795 on COVID-19 clinical outcomes, one (Balzanelli et al. 2022) and one (Verma et al. 2022) comparison were identified as the main heterogeneity contributor to COVID-19 susceptibility and severity. Notably, the recalculated results for susceptibility changed substantially after excluding those comparisons (please see Table 2 for more details).

### Publication Bias Test

Begg's test did not find any publication bias in the present study, which was confirmed by Egger's regression test.

### Risk of Bias/Quality Assessment

In an analysis of the risk bias of rs1800795 with IL-6 (Figure S4) and CRP (Figure S5) and rs1800629 with COVID-19 severity (Figure S6), the majority of studies (80–92.4%) presented with green color (Figure S4–S6), indicating a low risk of bias. In addition, no risk of bias was detected for rs1800629 with TNF-α (Figure S7) and CRP (Figure S8) and rs1800795 with COVID-19 severity (Figure S9). In summary, the current literature included is of high quality due to a low risk of bias (Figure S4–S9).

## Discussion

The A allele of rs1800629 significantly elevated TNF-α and CRP levels and increased COVID-19 severity and mortality. In contrast, the C allele of rs1800795 elevated IL-6 and CRP levels and increased COVID-19 susceptibility.

The up-regulated inflammatory parameters (Tables 1, 2, Fig. 2) associated with pro-inflammatory cytokines variants may be attributed to the increased transcriptional activity of IL-6 and TNF-α [33, 34]. Moreover, two plausible mechanisms can be proposed to explain the impacts of pro-inflammatory cytokines variants on COVID-19 clinical outcomes. (1) By inducing a cytokine storm. IL-6 and TNF-α are critical components of cytokine storm [37, 49–55]. The elevated TNF-α and IL-6 levels associated with pro-inflammatory cytokines variants (Tables 1, 2) may be helpful to the formation of cytokine storm, thus deteriorating COVID-19 outcomes (Tables 1, 2). (2) By inducing lymphocytopenia. Lymphocytes play a critical role in controlling SARS-CoV-2 infection [63]. A low abundance of CD8^+^ T and CD4^+^ T lymphocytes was associated with severe illness and high mortality of COVID-19 [64–66]. Therefore, the lymphocytopenia associated with rs1800629 [67] and rs1800795 [68] may worsen COVID-19 outcomes.

The A allele of rs1800629 vastly increased TNF-α and CRP levels (Table 1, Fig. 2), indicating that individuals with the rs1800629 A allele are at high risk of cytokine storm and may have poor outcomes for COVID-19. Intriguingly, this speculation was verified in the present study, whereas the rs1800629 A allele significantly increased the severity and mortality of COVID-19 (Table 1, Figs. 4, 5). Notably, the increased levels of IL-6 were recognized as a maker of severe COVID-19 [69–71]. However, rs1800795 did not impact the severity of COVID-19 despite elevating IL-6 levels (Table 2, Fig. 2, Figure S2), since only 381 individuals were included for analyzing the impact of rs1800795 on COVID-19 severity, which largely lowered the statistical power and needs to be confirmed by future clinical trials.

The susceptibility of COVID-19 was increased 1.59–1.88 fold in Caucasians with the rs1800629 A allele (Table 1), indicating that Caucasians with the rs1800629 A allele are at high risk of suffering COVID-19. In addition, the mortality of COVID-19 increased 3.17-fold in Caucasians with the rs1800629 A allele (Table 1), suggesting that Caucasians with the rs1800629 A allele are at high risk of death. The specific reason why the impacts of rs1800629 on COVID-19 susceptibility and mortality were significant in Caucasians rather than in Asians was likely that the distribution frequency of the A allele was much higher in Caucasian individuals with COVID-19 (Caucasian individuals with COVID-19 vs. Asian individuals with COVID-19 = [10.7–23.4%] vs. [1.8–7.3%]) [72, 73].

The present study showed that pro-inflammatory cytokines variants remodeled the systemic inflammatory profile and impacted COVID-19 outcomes. Since the cytokine storm was closely linked to COVID-19 outcomes [43–48], it indicated that the impacts of pro-inflammatory cytokines variants on COVID-19 outcomes (Tables 1, 2) were mediated, at least partly, by the impacts of pro-inflammatory cytokines variants on systemic inflammatory profile (Tables 1, 2). Since anti-TNF-α (e.g., infliximab) and anti-IL-6 (e.g., tocilizumab) therapies were effective in individuals with severe illness [74–76], it indicated that targeting TNF-α and IL-6 may help prevent COVID-19 progression in individuals with rs1800629 and rs1800795. Large-scale clinical trials are urgently needed to verify this hypothesis.

Moreover, according to Anastassopoulou et al. [23]. proposals, safe and effective vaccines should be given priority to individuals at high genetic risk of developing COVID-19. Since rs1800629 A allele and rs1800795 C allele significantly increased the risk of COVID-19 (Tables 1, 2, Fig. 3), it indicated that individuals with variants of rs1800795 and rs1800629 should be prioritized for vaccination against COVID-19. Genetic screening of rs1800795 and rs1800629 is necessary for the public to achieve this goal.

## Conclusions

The C allele of rs1800795 increased the risk of COVID-19 and plasma levels of IL-6 and CRP. In contrast, the A allele of rs1800629 increased the severity and mortality of COVID-19 and plasma levels of TNF-α and CRP. These results hint that rs1800629 and rs1800795 variants of pro-inflammatory cytokines have significant impacts on COVID-19 clinical outcomes and systemic inflammatory profile. rs1800629 may serve as a genetic marker for severe COVID-19.

### Supplementary Information

Below is the link to the electronic supplementary material.

Supplementary file1 (DOCX 7728 KB)

## Data Availability

All data generated or analyzed during this study are included in this published article and its Supplementary Material.
