# Supplementary material for: Impacts of Inflammatory Cytokines Variants on Systemic Inflammatory Profile and COVID-19 Severity
Source: J Epidemiol Glob Health. 2024 Feb 20;14(2):363–78. doi: 10.1007/s44197-024-00204-w (PMC11176143; doi:10.1007/s44197-024-00204-w)
Supplement: Supplementary file 1 — Supplementary file1 (DOCX 7728 KB) [file 44197_2024_204_MOESM1_ESM.docx]

*Table S1* **Characteristics of the included studies.**

| **First author, reference** | **Year** | **Country** | **Ethnicity** | **Gender** | **Genotyping Methods** | **Polymorphisms** | **Total sample size** |
| --- | --- | --- | --- | --- | --- | --- | --- |
| Ali HN [S1] | 2022 | Iraqi | Caucasian | M/F | RT-PCR | rs1800629 | 239 |
| Saleh A [S2] | 2022 | Egypt | Caucasian | M/F | RT-PCR | rs1800629 | 1084 |
| Heidari Nia [S3] | 2022 | Iran | Caucasian | M/F | RT-PCR | rs1800629 | 550 |
| Palacios Y [S4] | 2021 | Mexico | Mexican | M/F | RT-PCR | rs1800629 | 80 |
| Chong WP [S5] | 2022 | China | Asian | M/F | RT-PCR | rs1800629 | 925 |
| Wang S [S6] | 2022 | China | Asian | M/F | RT-PCR | rs1800629 | 208 |
| Minashkin MM [S7] | 2022 | Russia | Caucasian | M/F | RT-PCR | rs1800629 | 396 |
| Balzanelli MG [S8] | 2022 | Vietnam | Asian | M/F | RT-PCR | rs1800629 | 84 |
| Sotomayor-Lugo F [S9] | 2022 | Cuba | Caucasian | M/F | RT-PCR | rs1800629 | 1974 |
| Aladawy SA [S10] | 2022 | Egypt | Caucasian | M/F | RT-PCR | rs1800795 | 70 |
| Rodrigues FBB [S11] | 2022 | Brazil | Amazonian | M/F | RT-PCR | rs1800795 | 527 |
| Giannitrapani L [S12] | 2022 | Italy | Caucasian | M/F | RT-PCR | rs1800629 | 125 |
| Fricke-Galindo I [S13] | 2022 | Mexico | Mexican | M/F | Unspecified | rs1800629 | 138 |
| Reviono [S14] | 2022 | Indonesia | Asian | M/F | RT-PCR | rs1800629 | 1351 |
| Falahi S [S15] | 2022 | Iran | Caucasian | M/F | RT-PCR | rs1800795 | 36 |
| Kerget F [S16] | 2021 | Turkey | Turkish | M/F | RT-PCR | rs1800795 | 346 |
| Verma S [S17] | 2022 | India | Indian | M/F | RT-PCR | rs1800795 | 70 |
| Fishchuk L [S18] | 2021 | Ukraine | Caucasian | M/F | PCR-RFLP | rs1800629 | 31 |
| Smieszek SP [S19] | 2022 | UA | Caucasian | M/F | Unspecified | rs1800795 | 71 |
| Shaker OG [S20] | 2013 | Egypt | Caucasian | M/F | PCR | rs1800629 | 100 |
| Shaker OG [S20] | 2013 | Egypt | Caucasian | M/F | PCR | rs1800629 | 100 |
| Shaker OG [S20] | 2013 | Egypt | Caucasian | M/F | PCR | rs1800629 | 100 |
| Bounder G [S21] | 2020 | Morocco | Caucasian | M/F | PCR | rs1800629 | 218 |
| Vikram NK [S22] | 2011 | India | Indian | M/F | PCR–RFLP | rs1800629 | 151 |
| Banerjee N [S23] | 2011 | India | Indian | M/F | PCR | rs1800629 | 397 |
| Kim JM [S24] | 2017 | Korea | Asian | M/F | PCR–RFLP | rs1800629 | 286 |
| Daulay M [S25] | 2019 | Indonesia | Indonesian | M/F | PCR–RFLP | rs1800629 | 120 |
| Ivanova M [S26] | 2021 | Bulgaria | Caucasian | M/F | PCR | rs1800629 | 81 |
| Ivanova M [S26] | 2021 | Bulgaria | Caucasian | M/F | PCR–RFLP | rs1800629 | 215 |
| Divella R [S27] | 2019 | Italy | Caucasian | M/F | PCR–RFLP | rs1800629 | 165 |
| Jahid M [S28] | 2017 | India | Indian | M/F | PCR–RFLP | rs1800629 | 187 |
| Ghaderian SM [S29] | 2011 | Iran | Caucasian | M/F | RT-PCR | rs1800629 | 996 |
| Ghaderian SM [S29] | 2011 | Iran | Caucasian | M/F | RT-PCR | rs1800629 | 910 |
| Umare VD [S30] | 2017 | India | Indian | M/F | PCR–RFLP | rs1800629 | 200 |
| Liaquat A [S31] | 2014 | Pakistan | Caucasian | M/F | PCR | rs1800629 | 300 |
| Liaquat A [S31] | 2014 | Pakistan | Caucasian | M/F | PCR | rs1800629 | 250 |
| Toutouzas K [S32] | 2017 | Greece | Caucasian | M/F | PCR–RFLP | rs1800795 | 157 |
| Akhter MS [S33] | 2019 | India | Indian | M/F | PCR | rs1800795 | 200 |
| Pereira DS [S34] | 2013 | Brazil | Brazilian | F | PCR | rs1800795 | 449 |
| Sharma A [S35] | 2018 | India | Indian | M/F | PCR–RFLP | rs1800795 | 100 |
| Sharma A [S35] | 2018 | India | Indian | M/F | PCR–RFLP | rs1800795 | 100 |
| Kelberman D [S36] | 2004 | UK/Sweden/France/Italy | Caucasian | M/F | PCR–RFLP | rs1800795 | 193 |
| Kelberman D [S36] | 2004 | UK/Sweden/France/Italy | Caucasian | M/F | PCR–RFLP | rs1800795 | 218 |
| Kelberman D [S36] | 2004 | UK/Sweden/France/Italy | Caucasian | M/F | PCR–RFLP | rs1800795 | 165 |
| Kelberman D [S36] | 2004 | UK/Sweden/France/Italy | Caucasian | M/F | PCR–RFLP | rs1800795 | 198 |
| Marso SP [S37] | 2006 | USA | Caucasian | M/F | PCR | rs1800795 | 117 |
| Chmurzynska A [S38] | 2019 | Poland | Caucasian | F | RT-PCR | rs1800795 | 95 |
| Bhatt SP [S39] | 2018 | India | Indian | M/F | PCR–RFLP | rs1800795 | 171 |
| Bhatt SP [S39] | 2018 | India | Indian | M/F | PCR–RFLP | rs1800795 | 69 |
| Potaczek DP [S40] | 2006 | Poland | Caucasian | M/F | Unspecified | rs1800795 | 20 |
| Potaczek DP [S40] | 2006 | Poland | Caucasian | M/F | Unspecified | rs1800795 | 18 |
| Sie MP [S41] | 2008 | France | Caucasian | M/F | PCR | rs1800795 | 3849 |
| Biswas S [S42] | 2014 | India | Indian | M/F | PCR | rs1800795 | 500 |
| Biswas S [S42] | 2014 | India | Indian | M/F | PCR | rs1800795 | 814 |
| Bennet AM [S43] | 2003 | Sweden | Caucasian | M/F | PCR | rs1800795 | 1147 |
| Bennet AM [S43] | 2003 | Sweden | Caucasian | M/F | PCR | rs1800795 | 1500 |
| Bennermo M [S44] | 2011 | Sweden | Caucasian | M/F | RT-PCR | rs1800795 | 356 |
| Bennermo M [S44] | 2011 | Sweden | Caucasian | M/F | RT-PCR | rs1800795 | 378 |
| Basso F [S45] | 2002 | UK | Caucasian | M | PCR–RFLP | rs1800795 | 498 |
| Basso F [S45] | 2002 | UK | Caucasian | M | PCR–RFLP | rs1800795 | 1109 |
| Pramudji H [S46] | 2019 | Indonesia | Indonesian | M/F | PCR–RFLP | rs1800795 | 89 |
| Pramudji H [S46] | 2019 | Indonesia | Indonesian | M/F | PCR–RFLP | rs1800795 | 89 |
| Wernstedt I [S47] | 2004 | Sweden | Caucasian | M/F | PCR–RFLP | rs1800795 | 471 |

M: male; F: female; PCR: polymerase chain reaction; RT-PCR: reverse transcription-polymerase chain reaction; PCR-RFLP: polymerase chain

reaction-restriction fragment length polymorphism.

*Table S2* **Plasma TNF-α levels by the genotype of rs1800629.**

| **First author, reference** | **Number** | |  | **TNF-α, pg/mL** | |
| --- | --- | --- | --- | --- | --- |
|  | **GG** | **GA+AA** |  | **GG** | **GA+AA** |
| Shaker OG [S20] | 32 | 68 |  | 57.6±25.1 | 63.62±26.71 |
| Shaker OG [S20] | 24 | 76 |  | 50.6±15.3 | 52.71±17.71 |
| Shaker OG [S20] | 66 | 34 |  | 28.4±9.2 | 30.78±12.07 |
| Bounder G [S21] | 171 | 47 |  | 44.6±17.9 | 130.55±147.04 |
| Vikram NK [S22] | 119 | 32 |  | 57.5±27.2 | 50.7±26.8 |
| Banerjee N [S23] | 316 | 81 |  | 6.3±6.4 | 15.3±8.7 |
| Kim JM [S24] | 237 | 49 |  | 64.3±38.8 | 73.0±45.9 |
| Daulay M [S25] | 113 | 7 |  | 3.98±0.36 | 5.58±0.33 |
| Ivanova M [S26] | 74 | 7 |  | 12.19±20.9 | 14.77±20.9 |
| Ivanova M [S26] | 165 | 50 |  | 3.75±2.9 | 3.6±2.5 |
| Divella R [S27] | 55 | 110 |  | 88.5±45 | 148.2±27.15 |
| Jahid M [S28] | 108 | 79 |  | 35.5±11.00 | 37.16±11.45 |
| Ghaderian SM [S29] | 681 | 315 |  | 17.43±0.58 | 17.65±0.58 |
| Ghaderian SM [S29] | 717 | 193 |  | 11.76±0.52 | 11.72±0.48 |

TNF-α: tumor necrosis factor-α.

*Table S3* **Plasma CRP levels by the genotype of rs1800629.**

| **First author, reference** | **Number** | |  | **CRP, g/L** | |
| --- | --- | --- | --- | --- | --- |
|  | **GG** | **GA+AA** |  | **GG** | **GA+AA** |
| Umare VD [S30] | 128 | 72 |  | 10.1±9.6 | 17.6±13.6 |
| Liaquat A [S31] | 223 | 77 |  | 1.08±0.7 | 1.19±0.66 |
| Liaquat A [S31] | 72 | 178 |  | 2.70±3.4 | 4.92±4.96 |
| Vikram NK [S22] | 119 | 32 |  | 3.40±4.0 | 3.5±4.5 |
| Saleh A [S2] | 192 | 708 |  | 21.56±14.85 | 39.3±30.13 |
| Jahid M [S28] | 108 | 79 |  | 17.25 ± 7.12 | 18.72±6.91 |

CRP: C-reactive protein.

*Table S4* **Plasma IL-6 levels by the genotype of rs1800795.**

| **First author, reference** | **Number** | |  | **IL-6, pg/mL** | |
| --- | --- | --- | --- | --- | --- |
|  | **GG** | **GC+CC** |  | **GG** | **GC+CC** |
| Toutouzas K [S32] | 30 | 127 |  | 2.73±1.08 | 4.71±2.29 |
| Akhter MS [S33] | 118 | 82 |  | 15.17±10.95 | 22.33±13.41 |
| Pereira DS [S34] | 257 | 192 |  | 2.1±3.5 | 2.2±3.3 |
| Sharma A [S35] | 65 | 35 |  | 13.44±6.61 | 14.25±5.73 |
| Sharma A [S35] | 69 | 31 |  | 11.15±3.99 | 13.32±5.14 |
| Kelberman D [S36] | 76 | 117 |  | 1.83±1.22 | 1.67±1.11 |
| Kelberman D [S36] | 62 | 156 |  | 1.20±0.67 | 1.29±0.72 |
| Kelberman D [S36] | 87 | 78 |  | 2.2±1.47 | 2.22±1.48 |
| Kelberman D [S36] | 103 | 95 |  | 1.14±0.75 | 1.28±0.85 |
| Marso SP [S37] | 50 | 67 |  | 2.6±1.8 | 4.9±6.4 |
| Chmurzynska A [S38] | 24 | 71 |  | 2.20±1.56 | 3.03±3.49 |
| Bhatt SP [S39] | 113 | 58 |  | 14.2±9.2 | 21.53±7.11 |
| Bhatt SP [S39] | 44 | 25 |  | 14.4±7.9 | 17.14±8.86 |
| Potaczek DP [S40] | 10 | 10 |  | 1.72±0.34 | 3.21±0.46 |
| Potaczek DP [S40] | 5 | 13 |  | 2.08±0.62 | 2.78±0.4 |
| Sie MP [S41] | 1390 | 2459 |  | 2.1±1.5 | 2.27±1.73 |
| Biswas S [S42] | 348 | 152 |  | 12.60±33.54 | 28.09±108.71 |
| Biswas S [S42] | 407 | 407 |  | 7.28±6.71 | 10.1±55.63 |
| Bennet AM [S43] | 295 | 852 |  | 1.73±3.40 | 1.59±2.90 |
| Bennet AM [S43] | 398 | 1102 |  | 0.93±2.25 | 1.29±0.72 |
| Bennermo M [S44] | 119 | 237 |  | 0.81±1.87 | 0.79±1.72 |
| Bennermo M [S44] | 109 | 269 |  | 0.58±0.65 | 0.66±0.91 |

IL-6: interleukin-6.

*Table S5* **Plasma CRP levels by the genotype of rs1800795.**

| **First author, reference** | **Number** | |  | **CRP, g/L** | |
| --- | --- | --- | --- | --- | --- |
|  | **GG** | **GC+CC** |  | **GG** | **GC+CC** |
| Basso F [S45] | 161 | 337 |  | 4.28±5.35 | 3.67±4.21 |
| Basso F [S45] | 375 | 734 |  | 3.18±4.53 | 3.36±4.42 |
| Pramudji H [S46] | 5 | 84 |  | 1.19±0.58 | 2.33±2.18 |
| Pramudji H [S46] | 22 | 67 |  | 0.56±0.61 | 0.47±0.49 |
| Kelberman D [S36] | 76 | 117 |  | 2.41±2.86 | 2.34±2.77 |
| Kelberman D [S36] | 62 | 156 |  | 1.28±1.41 | 1.29±1.42 |
| Kelberman D [S36] | 87 | 78 |  | 2.18±2.07 | 2.44±2.32 |
| Kelberman D [S36] | 103 | 95 |  | 1.52±1.33 | 1.59±1.39 |
| Bhatt SP [S39] | 113 | 58 |  | 3.1±1.9 | 3.67±2.0 |
| Bhatt SP [S39] | 44 | 25 |  | 1.35±0.9 | 2.16±0.98 |
| Potaczek DP [S40] | 10 | 10 |  | 1.35±0.21 | 3.56±1.08 |
| Potaczek DP [S40] | 5 | 13 |  | 2.5±0.88 | 2.32±0.52 |
| Sie MP [S41] | 1390 | 2459 |  | 2.2±2.3 | 2.57±2.77 |
| Wernstedt I [S47] | 142 | 329 |  | 2.72±0.40 | 3.14±0.66 |
| Liaquat A [S31] | 182 | 68 |  | 3.69±4.4 | 5.85±4.98 |
| Liaquat A [S31] | 252 | 48 |  | 1.07±0.7 | 1.32±0.79 |

CRP: C-reactive protein.

*Table S6* **The genotype distribution frequency of rs1800629 in COVID-19 and non-COVID-19 individuals.**

| **First author, reference** | **Sample size**  **(case/control)** | **Case (N)** | | |  | **Control (N)** | | |
| --- | --- | --- | --- | --- | --- | --- | --- | --- |
|  |  | **GG** | **GA** | **AA** |  | **GG** | **GA** | **AA** |
| Ali HN [S1] | 125/114 | 87 | 37 | 1 |  | 93 | 17 | 4 |
| Saleh A [S2] | 900/184 | 192 | 288 | 420 |  | 84 | 40 | 60 |
| Heidari Nia [S3] | 275/275 | 104 | 135 | 36 |  | 76 | 139 | 60 |
| Palacios Y [S4] | 62/18 | 57 | 5 | 0 |  | 18 | 0 | 0 |
| Chong WP [S5] | 476/449 | 403 | 70 | 3 |  | 377 | 70 | 2 |
| Wang S [S6] | 75/133 | 67 | 7 | 1 |  | 127 | 6 | 0 |
| Minashkin MM [S7] | 318/78 | 233 | 79 | 6 |  | 64 | 14 | 0 |
| Balzanelli MG [S8] | 41/43 | 34 | 7 | 0 |  | 32 | 7 | 4 |
| Sotomayor-Lugo F [S9] | 1027/947 | 819 | 189 | 19 |  | 727 | 205 | 15 |

*Table S7* **The genotype distribution frequency of rs1800795 in COVID-19 and non-COVID-19 individuals.**

| **First author, reference** | **Sample size**  **(case/control)** | **Case (N)** | | |  | **Control (N)** | | |
| --- | --- | --- | --- | --- | --- | --- | --- | --- |
|  |  | **GG** | **GC** | **CC** |  | **GG** | **GC** | **CC** |
| Aladawy SA [S10] | 50/20 | 38 | 11 | 1 |  | 16 | 4 | 0 |
| Rodrigues FBB [S11] | 227/300 | 127 | 86 | 14 |  | 207 | 85 | 8 |
| Balzanelli MG [S8] | 41/43 | 29 | 11 | 1 |  | 14 | 23 | 6 |

*Table S8* **The genotype distribution frequency of rs1800629 in severe and non-severe COVID-19 individuals.**

| **First author, reference** | **Sample size**  **(case/control)** | **Severe COVID-19 (N)** | | |  | **Non-severe COVID-19 (N)** | | |
| --- | --- | --- | --- | --- | --- | --- | --- | --- |
|  |  | **GG** | **GA** | **AA** |  | **GG** | **GA** | **AA** |
| Ali HN [S1] | 52/73 | 36 | 16 | 0 |  | 51 | 21 | 1 |
| Saleh A [S2] | 456/444 | 0 | 120 | 336 |  | 192 | 168 | 84 |
| Heidari Nia [S3] | 179/96 | 71 | 83 | 25 |  | 33 | 52 | 11 |
| Giannitrapani L [S12] | 33/105 | 26 | 7 | 0 |  | 86 | 18 | 1 |
| Fricke-Galindo I [S13] | 976/375 | 901 | 74 | 1 |  | 353 | 22 | 0 |
| Palacios Y [S4] | 44/18 | 39 | 5 | 0 |  | 18 | 0 | 0 |
| Wang S [S6] | 24/30 | 20 | 4 | 0 |  | 27 | 2 | 1 |
| Minashkin MM [S7] | 222/176 | 154 | 64 | 4 |  | 145 | 30 | 1 |
| Sotomayor-Lugo F [S9] | 66/551 | 53 | 12 | 1 |  | 430 | 110 | 11 |
| Reviono [S14] | 19/17 | 18 | 1 | 0 |  | 17 | 0 | 0 |

*Table S9* **The genotype distribution frequency of rs1800795 in severe and non-severe COVID-19 individuals.**

| **First author, reference** | **Sample size**  **(case/control)** | **Severe COVID-19 (N)** | | |  | **Non-severe COVID-19 (N)** | | |
| --- | --- | --- | --- | --- | --- | --- | --- | --- |
|  |  | **GG** | **GC** | **CC** |  | **GG** | **GC** | **CC** |
| Aladawy SA [S10] | 38/12 | 30 | 8 | 0 |  | 8 | 3 | 1 |
| Falahi S [S15] | 175/171 | 106 | 57 | 12 |  | 103 | 54 | 14 |
| Giannitrapani L [S12] | 33/103 | 20 | 10 | 3 |  | 69 | 33 | 1 |
| Kerget F [S16] | 40/30 | 18 | 22 | 0 |  | 3 | 27 | 0 |
| Rodrigues FBB [S11] | 95/132 | 56 | 35 | 4 |  | 71 | 51 | 10 |
| Verma S [S17] | 97/145 | 66 | 31 | 0 |  | 120 | 25 | 0 |

*Table S10* **The genotype distribution frequency of rs1800629 in COVID-19 death and non-death individuals.**

| **First author, reference** | **Sample size**  **(case/control)** | **COVID-19 death (N)** | | |  | **Non-death (N)** | | |
| --- | --- | --- | --- | --- | --- | --- | --- | --- |
|  |  | **GG** | **GA** | **AA** |  | **GG** | **GA** | **AA** |
| Chong WP [S5] | 57/415 | 46 | 11 | 0 |  | 353 | 59 | 3 |
| Fishchuk L [S18] | 6/25 | 4 | 2 | 0 |  | 18 | 5 | 2 |
| Heidari Nia [S3] | 26/249 | 9 | 15 | 2 |  | 95 | 120 | 34 |
| Reviono [S14] | 3/46 | 3 | 0 | 0 |  | 45 | 1 | 0 |
| Saleh A [S2] | 60/840 | 0 | 0 | 60 |  | 192 | 288 | 360 |

*Table S11* **The genotype distribution frequency of rs1800795 in COVID-19 death and non-death individuals.**

| **First author, reference** | **Sample size**  **(case/control)** | **COVID-19 death (N)** | | |  | **Non-death (N)** | | |
| --- | --- | --- | --- | --- | --- | --- | --- | --- |
|  |  | **GG** | **GC** | **CC** |  | **GG** | **GC** | **CC** |
| Fishchuk L [S18] | 6/25 | 2 | 2 | 2 |  | 4 | 11 | 10 |
| Aladawy SA [S10] | 19/31 | 16 | 3 | 0 |  | 22 | 8 | 1 |
| Smieszek SP [S19] | 9/62 | 3 | 4 | 2 |  | 38 | 18 | 6 |

*Table S12* **Checklist of items to include when reporting a systematic review or meta-analysis.**

| **Section and Topic** | **Item #** | **Checklist item** | **Location where item is reported** |
| --- | --- | --- | --- |
| **TITLE** | | |  |
| Title | 1 | Identify the report as a systematic review. | 1 |
| **ABSTRACT** | | |  |
| Abstract | 2 | See the PRISMA 2020 for Abstracts checklist. | 2 |
| **INTRODUCTION** | | |  |
| Rationale | 3 | Describe the rationale for the review in the context of existing knowledge. | 3-5 |
| Objectives | 4 | Provide an explicit statement of the objective(s) or question(s) the review addresses. | 5 |
| **METHODS** | | |  |
| Eligibility criteria | 5 | Specify the inclusion and exclusion criteria for the review and how studies were grouped for the syntheses. | 5,6 |
| Information sources | 6 | Specify all databases, registers, websites, organisations, reference lists and other sources searched or consulted to identify studies. Specify the date when each source was last searched or consulted. | 6 |
| Search strategy | 7 | Present the full search strategies for all databases, registers and websites, including any filters and limits used. | 6 |
| Selection process | 8 | Specify the methods used to decide whether a study met the inclusion criteria of the review, including how many reviewers screened each record and each report retrieved, whether they worked independently, and if applicable, details of automation tools used in the process. | 6 |
| Data collection process | 9 | Specify the methods used to collect data from reports, including how many reviewers collected data from each report, whether they worked independently, any processes for obtaining or confirming data from study investigators, and if applicable, details of automation tools used in the process. | 7 |
| Data items | 10a | List and define all outcomes for which data were sought. Specify whether all results that were compatible with each outcome domain in each study were sought (e.g. for all measures, time points, analyses), and if not, the methods used to decide which results to collect. | 7 |
|  | 10b | List and define all other variables for which data were sought (e.g. participant and intervention characteristics, funding sources). Describe any assumptions made about any missing or unclear information. | 6 |
| Study risk of bias assessment | 11 | Specify the methods used to assess risk of bias in the included studies, including details of the tool(s) used, how many reviewers assessed each study and whether they worked independently, and if applicable, details of automation tools used in the process. | 9 |
| Effect measures | 12 | Specify for each outcome the effect measure(s) (e.g. risk ratio, mean difference) used in the synthesis or presentation of results. | 8 |
| Synthesis methods | 13a | Describe the processes used to decide which studies were eligible for each synthesis (e.g. tabulating the study intervention characteristics and comparing against the planned groups for each synthesis (item #5)). | 8 |
|  | 13b | Describe any methods required to prepare the data for presentation or synthesis, such as handling of missing summary statistics, or data conversions. | 8 |
|  | 13c | Describe any methods used to tabulate or visually display results of individual studies and syntheses. | 8 |
|  | 13d | Describe any methods used to synthesize results and provide a rationale for the choice(s). If meta-analysis was performed, describe the model(s), method(s) to identify the presence and extent of statistical heterogeneity, and software package(s) used. | 8 |
|  | 13e | Describe any methods used to explore possible causes of heterogeneity among study results (e.g. subgroup analysis, meta-regression). | 9 |
|  | 13f | Describe any sensitivity analyses conducted to assess robustness of the synthesized results. | 9 |
| Reporting bias assessment | 14 | Describe any methods used to assess risk of bias due to missing results in a synthesis (arising from reporting biases). | 9 |
| Certainty assessment | 15 | Describe any methods used to assess certainty (or confidence) in the body of evidence for an outcome. | 9 |
| **RESULTS** | | |  |
| Study selection | 16a | Describe the results of the search and selection process, from the number of records identified in the search to the number of studies included in the review, ideally using a flow diagram. | 10 |
|  | 16b | Cite studies that might appear to meet the inclusion criteria, but which were excluded, and explain why they were excluded. | 10 |
| Study characteristics | 17 | Cite each included study and present its characteristics. | 10 |
| Risk of bias in studies | 18 | Present assessments of risk of bias for each included study. | 13 |
| Results of individual studies | 19 | For all outcomes, present, for each study: (a) summary statistics for each group (where appropriate) and (b) an effect estimate and its precision (e.g. confidence/credible interval), ideally using structured tables or plots. | 11-13 |
| Results of syntheses | 20a | For each synthesis, briefly summarise the characteristics and risk of bias among contributing studies. | 11-13 |
|  | 20b | Present results of all statistical syntheses conducted. If meta-analysis was done, present for each the summary estimate and its precision (e.g. confidence/credible interval) and measures of statistical heterogeneity. If comparing groups, describe the direction of the effect. | 11-13 |
|  | 20c | Present results of all investigations of possible causes of heterogeneity among study results. | 13 |
|  | 20d | Present results of all sensitivity analyses conducted to assess the robustness of the synthesized results. | 13 |
| Reporting biases | 21 | Present assessments of risk of bias due to missing results (arising from reporting biases) for each synthesis assessed. | 13 |
| Certainty of evidence | 22 | Present assessments of certainty (or confidence) in the body of evidence for each outcome assessed. | 13 |
| **DISCUSSION** | | |  |
| Discussion | 23a | Provide a general interpretation of the results in the context of other evidence. | 14 |
|  | 23b | Discuss any limitations of the evidence included in the review. | 16 |
|  | 23c | Discuss any limitations of the review processes used. | 16 |
|  | 23d | Discuss implications of the results for practice, policy, and future research. | 16 |
| **OTHER INFORMATION** | | |  |
| Registration and protocol | 24a | Provide registration information for the review, including register name and registration number, or state that the review was not registered. | - |
|  | 24b | Indicate where the review protocol can be accessed, or state that a protocol was not prepared. | - |
|  | 24c | Describe and explain any amendments to information provided at registration or in the protocol. | - |
| Support | 25 | Describe sources of financial or non-financial support for the review, and the role of the funders or sponsors in the review. | 16 |
| Competing interests | 26 | Declare any competing interests of review authors. | 17 |
| Availability of data, code and other materials | 27 | Report which of the following are publicly available and where they can be found: template data collection forms; data extracted from included studies; data used for all analyses; analytic code; any other materials used in the review. | 17 |

**
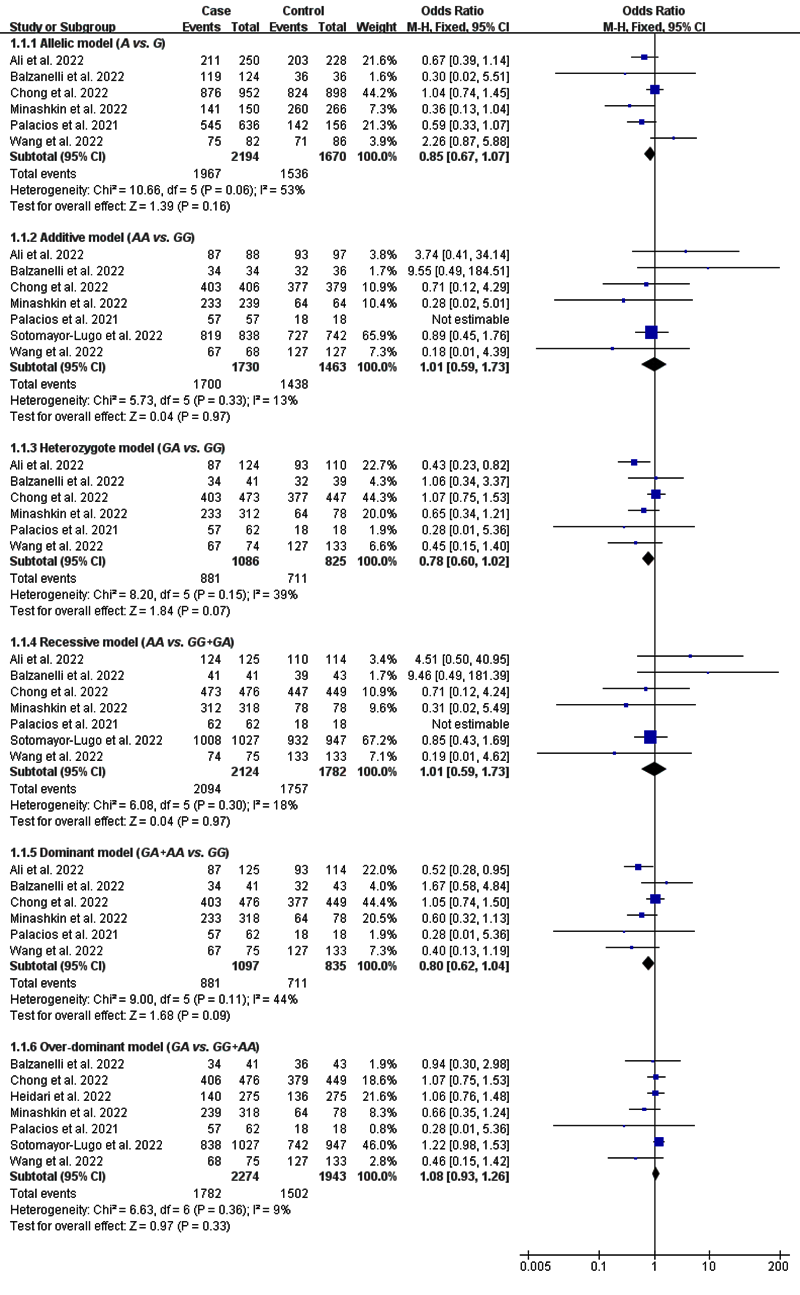
**

*Figure S1* **Forest plot of the meta-analysis between rs1800629 and COVID-19 susceptibility.**

**
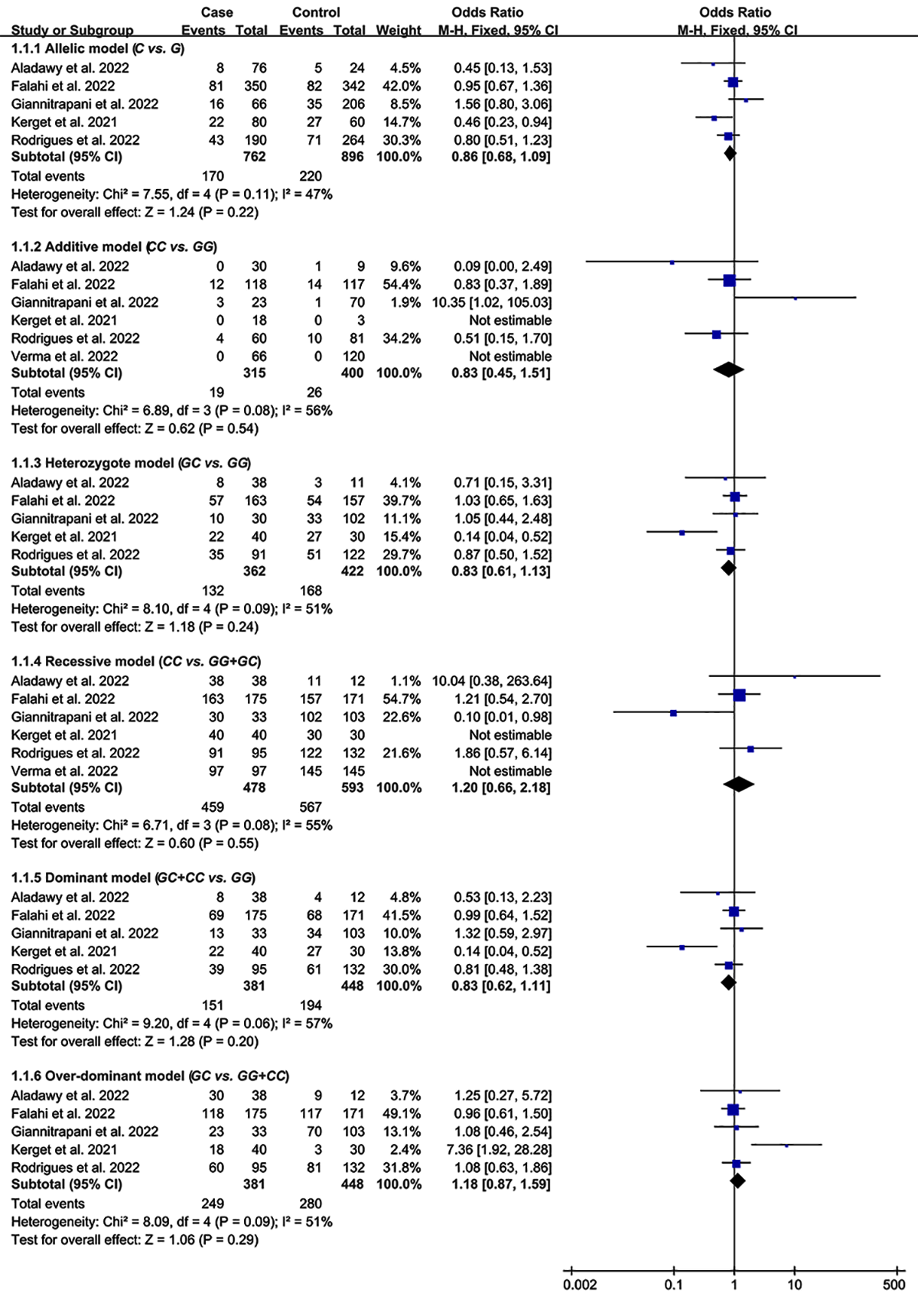
**

*Figure S2* **Forest plot of the meta-analysis between rs1800795 and COVID-19 severity.**

**
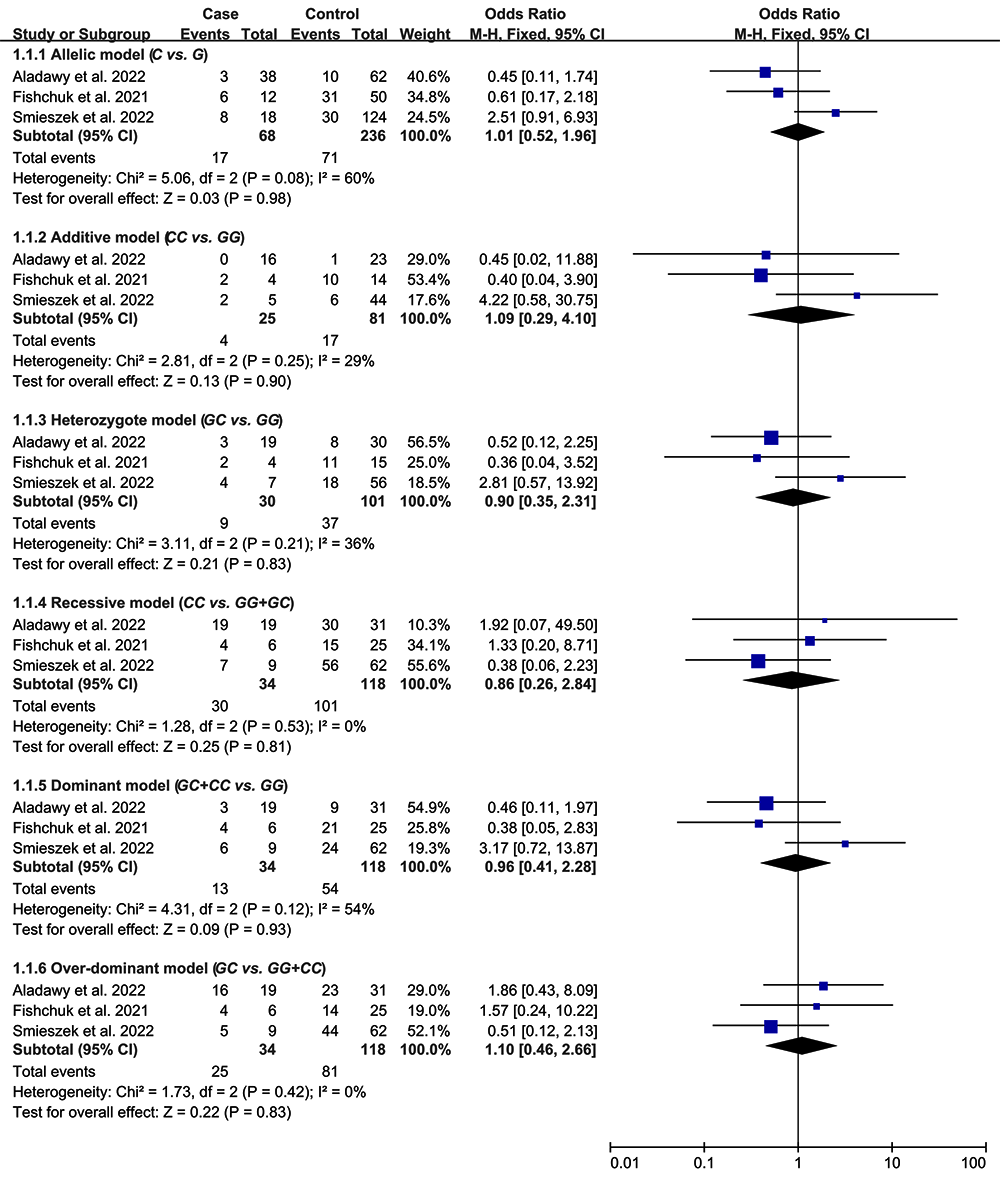
**

*Figure S3* **Forest plot of the meta-analysis between rs1800795 and COVID-19 mortality.**

**
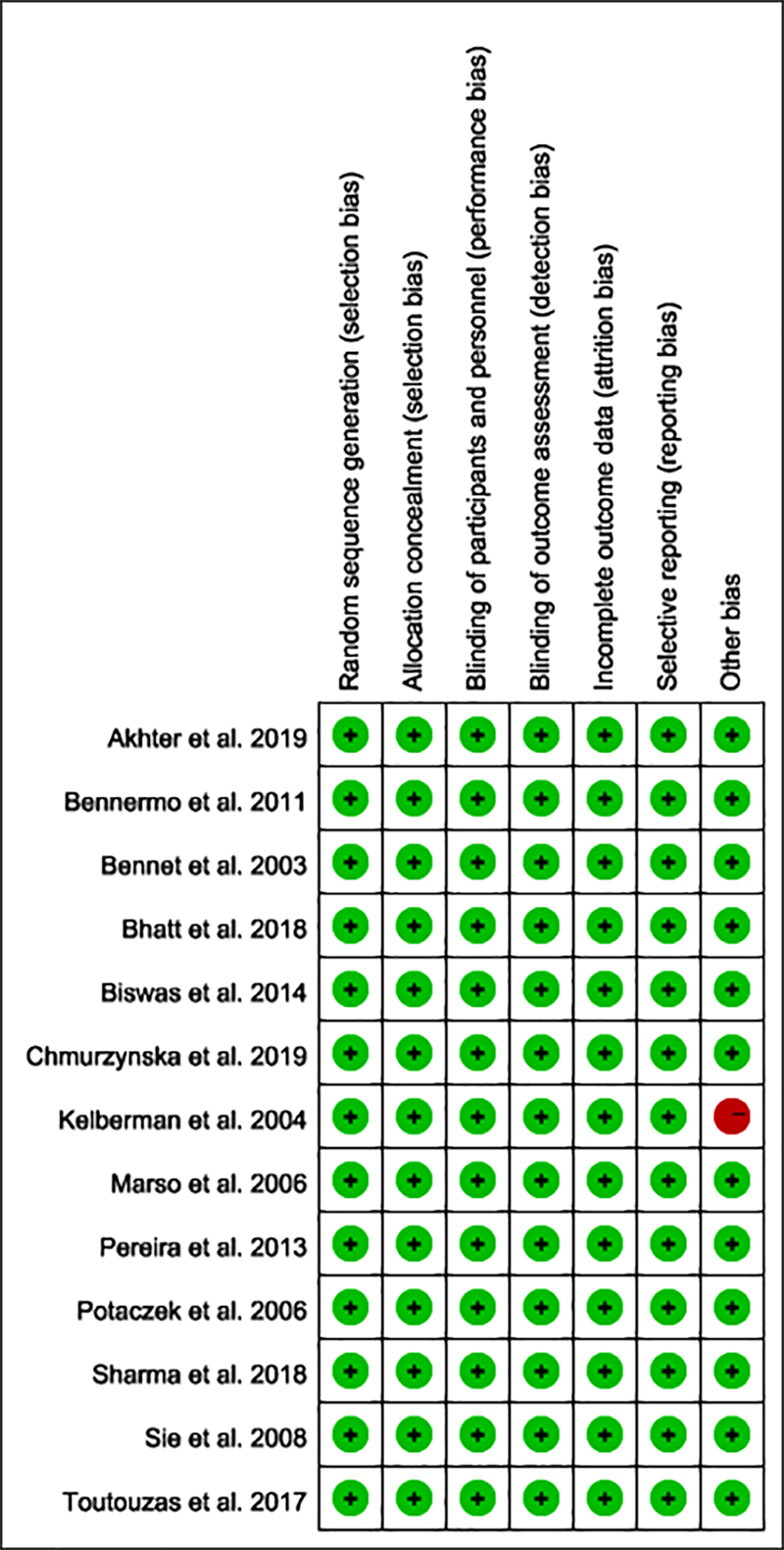
**

*Figure S4* **Risk bias plot of the meta-analysis between rs1800795 and interleukin-6 levels (for assessment of each entry, green represents low risk of bias and red refers to high risk of bias).**

**
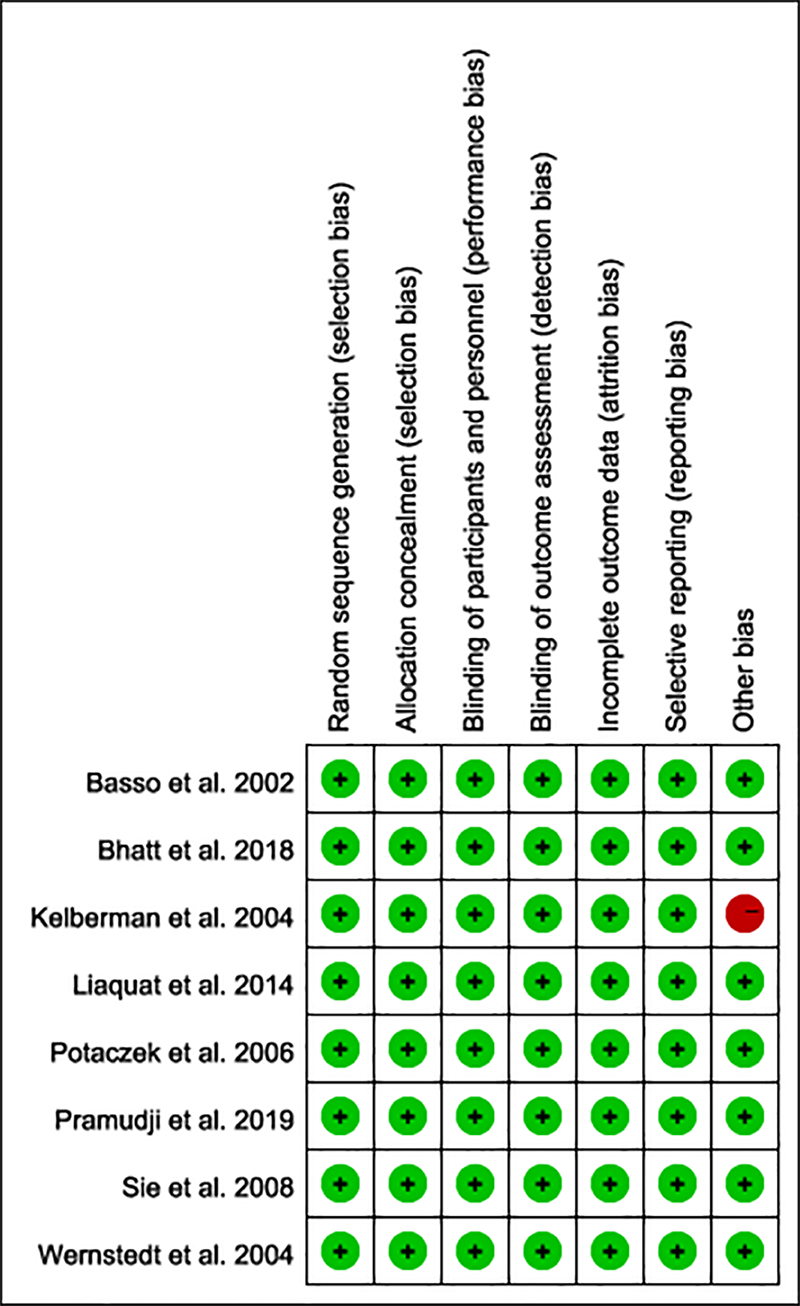
**

*Figure S5* **Risk bias plot of the meta-analysis between rs1800795 and C-reactive protein levels (for assessment of each entry, green represents low risk of bias and red refers to high risk of bias).**

**
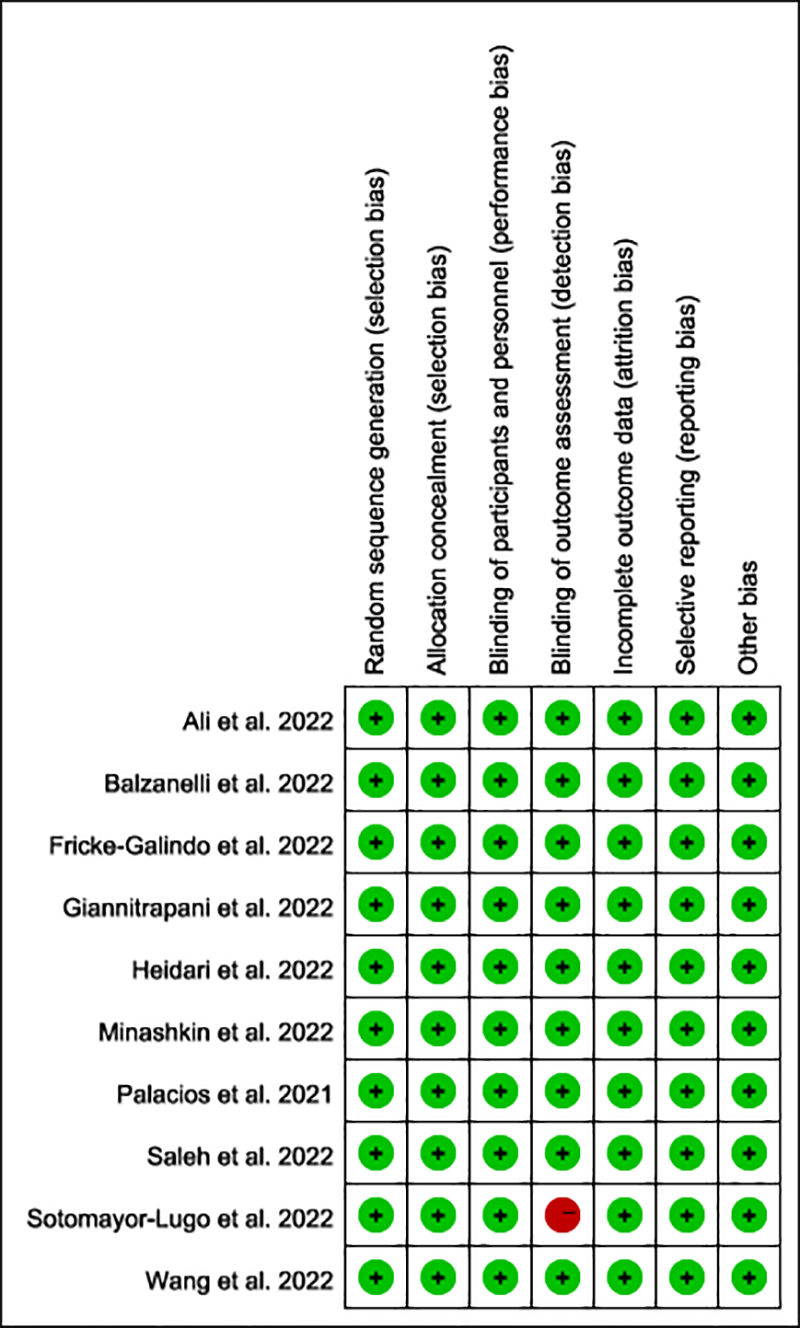
**

*Figure S6* **Risk bias plot of the meta-analysis between rs1800629 with COVID-19 severity (for assessment of each entry, green represents low risk of bias and red refers to high risk of bias).**

**
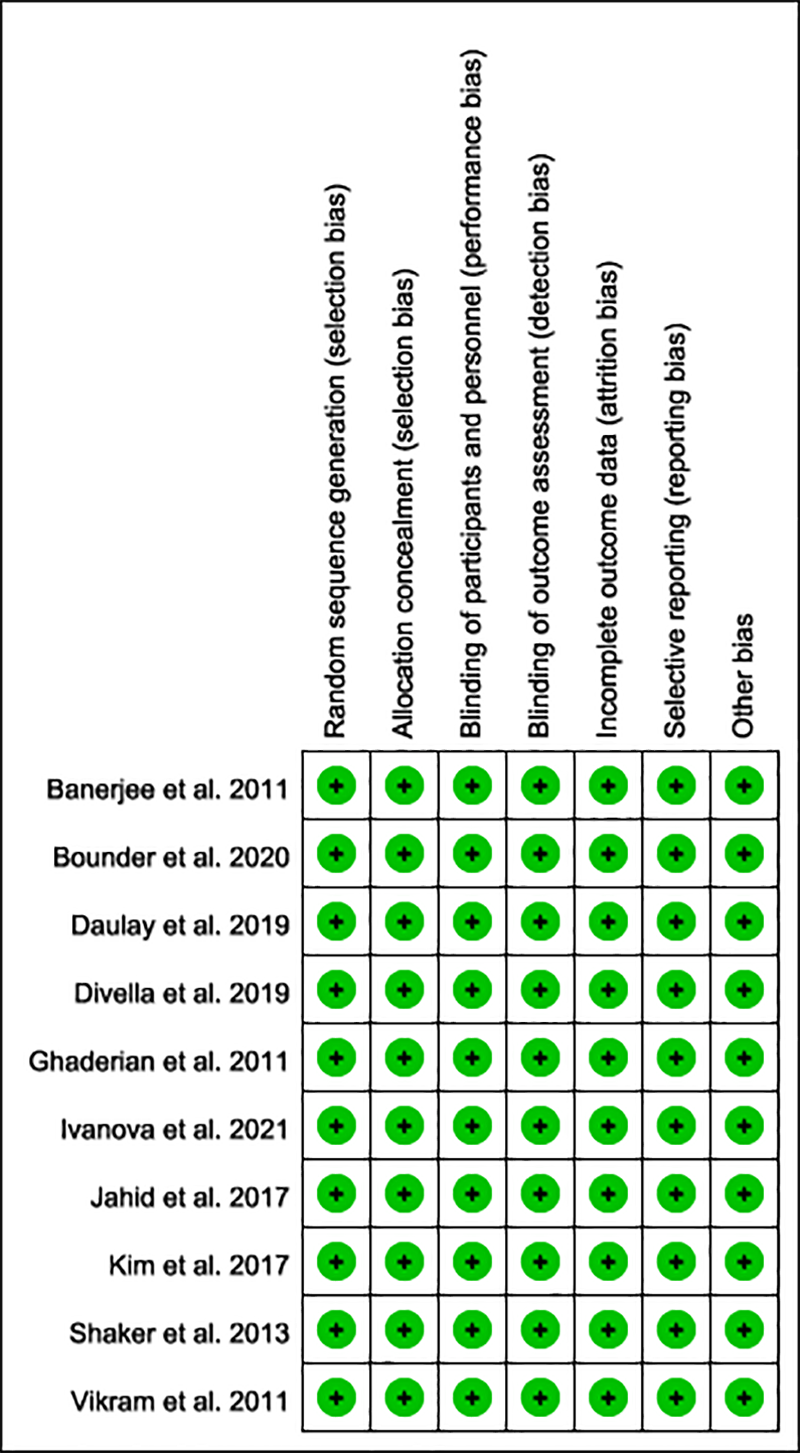
**

*Figure S7* **Risk bias plot of the meta-analysis between rs1800629 with tumor necrosis factor-α(for assessment of each entry, green represents low risk of bias and red refers to high risk of bias).**

**
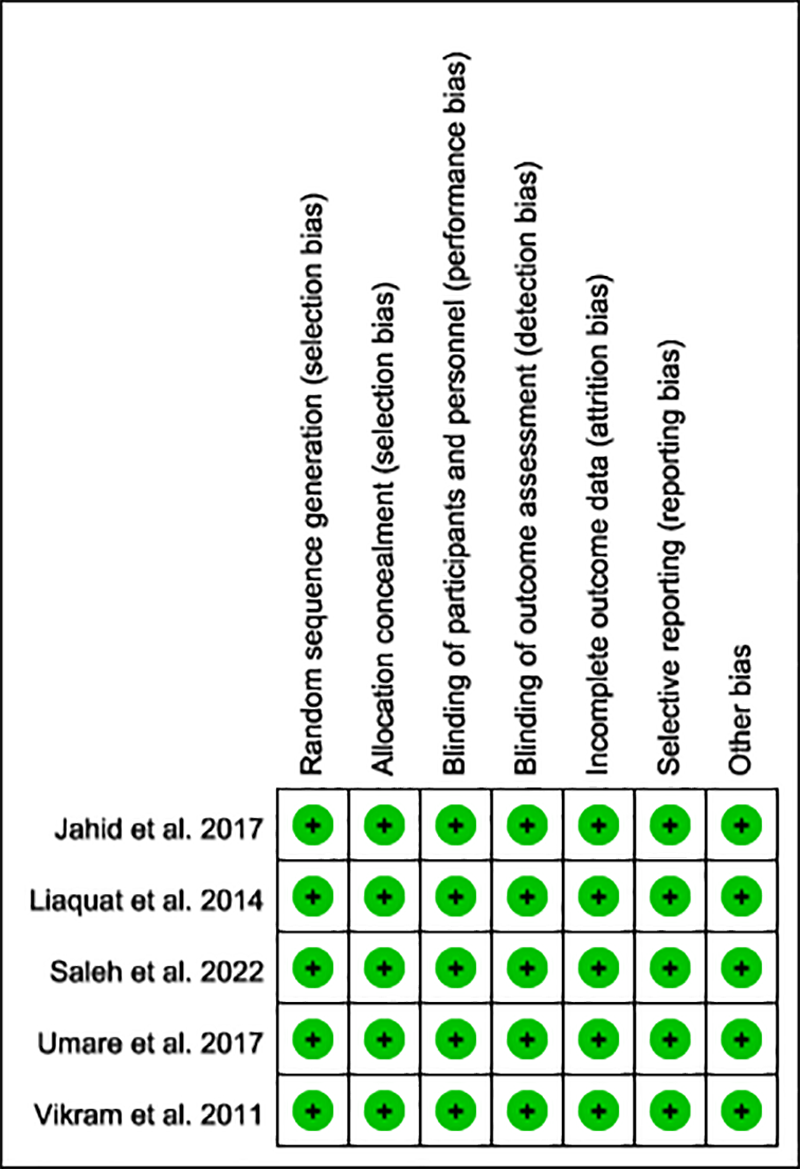
**

*Figure S8* **Risk bias plot of the meta-analysis between rs1800629 with C-reactive protein levels (for assessment of each entry, green represents low risk of bias and red refers to high risk of bias).**

**
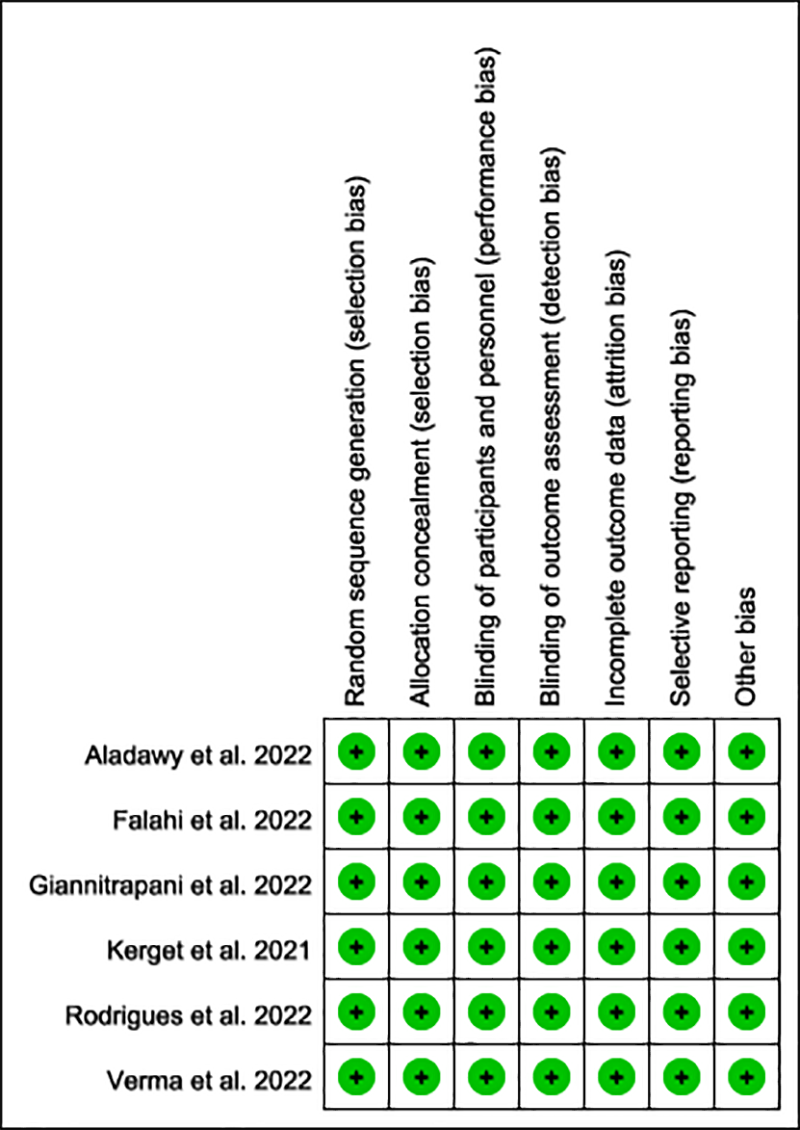
**

*Figure S9* **Risk bias plot of the meta-analysis between rs1800795 with COVID-19 severity (for assessment of each entry, green represents low risk of bias and red refers to high risk of bias).**

**References**

1. Ali HN, Niranji SS, Al-Jaf SMA. Association of tumor necrosis factor alpha -308 single nucleotide polymorphism with SARS CoV-2 infection in an Iraqi Kurdish population. J Clin Lab Anal. 2022;36(5):e24400. doi: 10.1002/jcla.24400.
2. Saleh A, Sultan A, Elashry MA, Farag A, Mortada MI, Ghannam MA, Saed AM, Ghoneem E. Association of TNF-α G-308 a Promoter Polymorphism with the Course and Outcome of COVID-19 Patients. Immunol Invest. 2022;51(3):546-557. doi: 10.1080/08820139.2020.1851709.
3. Heidari Nia M, Rokni M, Mirinejad S, Kargar M, Rahdar S, Sargazi S, Sarhadi M, Saravani R. Association of polymorphisms in tumor necrosis factors with SARS-CoV-2 infection and mortality rate: A case-control study and in silico analyses. J Med Virol. 2022;94(4):1502-1512. doi: 10.1002/jmv.27477.
4. Palacios Y, Ruiz A, Ramón-Luing LA, Ocaña-Guzman R, Barreto-Rodriguez O, Sánchez-Monciváis A, Tecuatzi-Cadena B, Regalado-García AG, Pineda-Gudiño RD, García-Martínez A, Juárez-Hernández F, Farias-Contreras JP, Fricke-Galindo I, Pérez-Rubio G, Falfán-Valencia R, Buendia-Roldan I, Medina-Quero K, Chavez-Galan L. Severe COVID-19 Patients Show an Increase in Soluble TNFR1 and ADAM17, with a Relationship to Mortality. Int J Mol Sci. 2021;22(16):8423. doi: 10.3390/ijms22168423.
5. Chong WP, Ip WK, Tso GH, Ng MW, Wong WH, Law HK, Yung RW, Chow EY, Au KL, Chan EY, Lim W, Peiris JS, Lau YL. The interferon gamma gene polymorphism +874 A/T is associated with severe acute respiratory syndrome. BMC Infect Dis. 2006;6:82. doi: 10.1186/1471-2334-6-82.
6. Wang S, Wei M, Han Y, Zhang K, He L, Yang Z, Su B, Zhang Z, Hu Y, Hui W. Roles of TNF-alpha gene polymorphisms in the occurrence and progress of SARS-Cov infection: a case-control study. BMC Infect Dis. 2008;8:27. doi: 10.1186/1471-2334-8-27.
7. Minashkin MM, Grigortsevich NY, Kamaeva AS, Barzanova VV, Traspov AA, Godkov MA, Ageev FA, Petrikov SS, Pozdnyakova NV. The Role of Genetic Factors in the Development of Acute Respiratory Viral Infection COVID-19: Predicting Severe Course and Outcomes. Biomedicines. 2022;10(3):549. doi: 10.3390/biomedicines10030549.
8. Balzanelli MG, Distratis P, Lazzaro R, Pham VH, Tran TC, Dipalma G, Bianco A, Serlenga EM, Aityan SK, Pierangeli V, Nguyen KCD, Inchingolo F, Tomassone D, Isacco CG. Analysis of Gene Single Nucleotide Polymorphisms in COVID-19 Disease Highlighting the Susceptibility and the Severity towards the Infection. Diagnostics (Basel). 2022;12(11):2824. doi: 10.3390/diagnostics12112824.
9. Sotomayor-Lugo F. The role of tumor necrosis factor alpha −308A>G polymorphism on the clinical states of SARS‑CoV‑2 infection. Egyptian Journal of Medical Human Genetics. 2022;23:55. <https://doi.org/10.1186/s43042-022-00274-0.>
10. Aladawy SA, Adel LA, Abdel Salam SA, Raafat RH, Khattab MA. Polymorphism in promotor region of IL6 gene as a predictor for severity in COVID-19 patients. Egypt J Immunol. 2022;29(2):1-9.
11. Rodrigues FBB. Association of polymorphisms of IL-6 pathway genes ( IL6, IL6R and IL6ST ) with COVID-19 severity in an Amazonian population. 2022. bioRxiv preprint doi: <https://doi.org/10.1101/2022.09.21.508870.>
12. Giannitrapani L, Augello G, Mirarchi L, Amodeo S, Veronese N, Sasso BL, Giglio RV, Licata A, Barbagallo M, Ciaccio M, Cervello M, Soresi M. Outcome predictors in SARS-CoV-2 disease (COVID-19): The prominent role of IL-6 levels and an IL-6 gene polymorphism in a western Sicilian population. J Infect. 2022;85(2):174-211. doi: 10.1016/j.jinf.2022.04.043.
13. Fricke-Galindo I, Buendía-Roldán I, Ruiz A, Palacios Y, Pérez-Rubio G, de Jesus Hernández-Zenteno R, Reyes-Melendres F, Zazueta-Márquez A, Alarcón-Dionet A, Guzmán-Vargas J, Bravo-Gutiérrez OA, Quintero-Puerta T, Gutiérrez-Pérez IA, Nava-Quiroz KJ, Bañuelos-Flores JL, Mejía M, Rojas-Serrano J, Ramos-Martínez E, Guzmán-Guzmán IP, Chávez-Galán L, Falfán-Valencia R. TNFRSF1B and TNF Variants Are Associated With Differences in Levels of Soluble Tumor Necrosis Factor Receptors in Patients With Severe COVID-19. J Infect Dis. 2022;226(5):778-787. doi: 10.1093/infdis/jiac101.
14. Reviono. Frequency of Interleukin-6 rs 1800796 (-572G/C) and 2069837 (intron 2A/G), TNF-αrs1800750 (-376G/A), and 1800629 (-308G/A) polymorphism in COVID-19 patients with clinical degrees in Central Java. Bali Medical Journal (Bali MedJ). 2022;11:1364-1368.
15. Falahi S, Zamanian MH, Feizollahi P, Rezaiemanesh A, Salari F, Mahmoudi Z, Gorgin Karaji A. Evaluation of the relationship between IL-6 gene single nucleotide polymorphisms and the severity of COVID-19 in an Iranian population. Cytokine. 2022;154:155889. doi: 10.1016/j.cyto.2022.155889.
16. Kerget F, Kerget B. Frequency of Interleukin-6 rs1800795 (-174G/C) and rs1800797 (-597G/A) Polymorphisms in COVID-19 Patients in Turkey Who Develop Macrophage Activation Syndrome. Jpn J Infect Dis. 2021;74(6):543-548. doi: 10.7883/yoken.JJID.2021.046.
17. Verma S, Verma S, Khan FH, Siddiqi Z, Raza ST, Abbas M, Mahdi F. Genetic polymorphisms of IL6 gene -174G > C and -597G > A are associated with the risk of COVID-19 severity. Int J Immunogenet. 2022. doi: 10.1111/iji.12605.
18. Fishchuk L, Rossokha Z, Pokhylko V, Cherniavska Y, Tsvirenko S, Kovtun S, Medvedieva N, Vershyhora V, Gorovenko N. Modifying effects of TNF-α, IL-6 and VDR genes on the development risk and the course of COVID-19. Pilot study. Drug Metab Pers Ther. 2021;37(2):133-139. doi: 10.1515/dmpt-2021-0127.
19. Smieszek SP, Przychodzen BP, Polymeropoulos VM, Polymeropoulos CM, Polymeropoulos MH. Assessing the potential correlation of polymorphisms in the IL6R with relative IL6 elevation in severely ill COVID-19 patients'. Cytokine. 2021;148:155662. doi: 10.1016/j.cyto.2021.155662.
20. Shaker OG, Sadik NA, El-Hamid NA. Impact of single nucleotide polymorphism in tumor necrosis factor-αgene 308G/A in Egyptian asthmatic children and wheezing infants. Hum Immunol. 2013;74(6):796-802. doi: 10.1016/j.humimm.2013.01.004.
21. Bounder G, Jouimyi MR, Boura H, Touati E, Michel V, Badre W, Jouhadi H, Kadi M, Eljihad M, Benomar H, Kettani A, Lebrazi H, Maachi F. Associations of the -238(G/A) and -308(G/A) TNF-αPromoter Polymorphisms and TNF-αSerum Levels with the Susceptibility to Gastric Precancerous Lesions and Gastric Cancer Related to Helicobacter pylori Infection in a Moroccan Population. Asian Pac J Cancer Prev. 2020;21(6):1623-1629. doi: 10.31557/APJCP.2020.21.6.1623.
22. Vikram NK, Bhatt SP, Bhushan B, Luthra K, Misra A, Poddar PK, Pandey RM, Guleria R. Associations of -308G/A polymorphism of tumor necrosis factor (TNF)-α gene and serum TNF-α levels with measures of obesity, intra-abdominal and subcutaneous abdominal fat, subclinical inflammation and insulin resistance in Asian Indians in north India. Dis Markers. 2011;31(1):39-46. doi: 10.3233/DMA-2011-0802.
23. Banerjee N, Nandy S, Kearns JK, Bandyopadhyay AK, Das JK, Majumder P, Basu S, Banerjee S, Sau TJ, States JC, Giri AK. Polymorphisms in the TNF-α and IL10 gene promoters and risk of arsenic-induced skin lesions and other nondermatological health effects. Toxicol Sci. 2011;121(1):132-9. doi: 10.1093/toxsci/kfr046.
24. Kim JM, Kang HJ, Kim JW, Bae KY, Kim SW, Kim JT, Park MS, Cho KH. Associations of Tumor Necrosis Factor-αand Interleukin-1βLevels and Polymorphisms with Post-Stroke Depression. Am J Geriatr Psychiatry. 2017;25(12):1300-1308. doi: 10.1016/j.jagp.2017.07.012.
25. Daulay M, Sari MI, Wahyuni DD, Syarifah S. The Gene Polymorphisms (-308G/A) and the Tumor Necrosis Factor-alpha Levels in Type 2 Diabetic Patients with and Without Tuberculosis Infection. Open Access Maced J Med Sci. 2019;7(23):3960-3964. doi: 10.3889/oamjms.2019.824.
26. Ivanova M, Manolova I, Stoilov R, Stanilova S. The synergistic role of TNFA - 308G/A and IL10 - 1082A/G polymorphisms in ankylosing spondylitis. Rheumatol Int. 2021;41(12):2215-2224. doi: 10.1007/s00296-021-04984-3.
27. Divella R, Daniele A, DE Luca R, Mazzocca A, Ruggieri E, Savino E, Casamassima P, Simone M, Sabba C, Paradiso A. Synergism of Adipocytokine Profile and ADIPOQ/TNF-α Polymorphisms in NAFLD-associated MetS Predict Colorectal Liver Metastases Outgrowth. Cancer Genomics Proteomics. 2019;16(6):519-530. doi: 10.21873/cgp.20154.
28. Jahid M, Rehan-Ul-Haq, Jha PK, Chawla D, Avasthi R, Ahmed RS. Tumor necrosis factor-α-308 polymorphism in North Indian rheumatoid arthritis patients and association with mRNA and serum TNF-α. Clin Rheumatol. 2017;36(10):2209-2216. doi: 10.1007/s10067-017-3774-7.
29. Ghaderian SM, Akbarzadeh Najar R, Tabatabaei Panah AS. Tumor necrosis factor-α: investigation of gene polymorphism and regulation of TACE-TNF-α system in patients with acute myocardial infarction. Mol Biol Rep. 2011;38(8):4971-7. doi: 10.1007/s11033-010-0641-x.
30. Umare VD, Pradhan VD, Rajadhyaksha AG, Patwardhan MM, Ghosh K, Nadkarni AH. Impact of TNF-α and LTαgene polymorphisms on genetic susceptibility in Indian SLE patients. Hum Immunol. 2017;78(2):201-208. doi: 10.1016/j.humimm.2016.11.002.
31. Liaquat A, Asifa GZ, Zeenat A, Javed Q. Polymorphisms of tumor necrosis factor-alpha and interleukin-6 gene and C-reactive protein profiles in patients with idiopathic dilated cardiomyopathy. Ann Saudi Med. 2014;34(5):407-14. doi: 10.5144/0256-4947.2014.407.
32. Toutouzas K, Klettas D, Anousakis-Vlachochristou N, Melidis K, Azilazian Z, Asimomiti M, Karanasos A, Spanos A, Tsiamis E, Nihoyannopoulos P, Tousoulis D. The -174 G>C Interleukin-6 Gene Polymorphism is Associated with Angiographic Progression of Coronary Artery Disease over a 4-Year Period. Hellenic J Cardiol. 2017;58(1):80-86. doi: 10.1016/j.hjc.2017.02.002.
33. Akhter MS, Biswas A, Abdullah SM, Hobani Y, Ranjan R, Behari M, Saxena R. Influence of Interleukin-6 (IL-6) Promoter Gene Polymorphisms (-174G>C, -572G>C, and -597G>A) on IL-6 Plasma Levels and Their Impact in the Development of Acute Ischemic Stroke in Young Indians. Clin Appl Thromb Hemost. 2019;25:1076029619854136. doi: 10.1177/1076029619854136.
34. Pereira DS, Mateo EC, de Queiroz BZ, Assumpção AM, Miranda AS, Felício DC, Rocha NP, da Cruz dos Anjos DM, Pereira DA, Teixeira AL, Pereira LS. TNF-α, IL6, and IL10 polymorphisms and the effect of physical exercise on inflammatory parameters and physical performance in elderly women. Age (Dordr). 2013;35(6):2455-63. doi: 10.1007/s11357-013-9515-1.
35. Sharma A, Singh K, Biswas A, Ranjan R, Kishor K, Pandey H, Kumar R, Mahapatra M, Oldenburg J, Saxena R. Impact of interleukin 6 promoter polymorphisms (-174 G > C, -572 G > C and -597 G > A) on plasma IL-6 levels and their influence on the development of DVT: a study from India. Hematology. 2018;23(10):833-838. doi: 10.1080/10245332.2018.1483546.
36. Kelberman D, Hawe E, Luong LA, Mohamed-Ali V, Lundman P, Tornvall P, Aillaud MF, Juhan-Vague I, Yudkin JS, Margaglione M, di Minno G, Tremoli E, Humphries SE; HIFMECH study group. Effect of Interleukin-6 promoter polymorphisms in survivors of myocardial infarction and matched controls in the North and South of Europe. The HIFMECH Study. Thromb Haemost. 2004;92(5):1122-8. doi: 10.1160/TH04-04-0202.
37. Marso SP, House JA, Hopkins PJ. Increase in interleukin-6 following arterial injury is related to insulin resistance, the -174G-->C polymorphism and complex plaque morphology. Int J Immunogenet. 2006;33(5):347-54. doi: 10.1111/j.1744-313X.2006.00622.x.
38. Chmurzynska A, Muzsik A, Krzyżanowska-Jankowska P, Walkowiak J, Bajerska J. The Effect of Habitual Fat Intake, IL6 Polymorphism, and Different Diet Strategies on Inflammation in Postmenopausal Women with Central Obesity. Nutrients. 2019;11(7):1557. doi: 10.3390/nu11071557.
39. Bhatt SP, Guleria R, Vikram NK, Vivekanandhan S, Singh Y, Gupta AK. Association of inflammatory genes in obstructive sleep apnea and non alcoholic fatty liver disease in Asian Indians residing in north India. PLoS One. 2018;13(7):e0199599. doi: 10.1371/journal.pone.0199599.
40. Potaczek DP, Undas A, Celinska-Lowenhoff M, Szczeklik A. The I allele of the angiotensin-converting enzyme gene polymorphism may determine an increase in homocysteine levels in fibrate-treated subjects. Cardiovasc Drugs Ther. 2006;20(3):229-32. doi: 10.1007/s10557-006-8374-8.
41. Sie MP, Mattace-Raso FU, Uitterlinden AG, Arp PP, Hofman A, Pols HA, Hoeks AP, Reneman RS, Asmar R, van Duijn CM, Witteman JC. The interleukin-6-174 G/C promoter polymorphism and arterial stiffness; the Rotterdam Study. Vasc Health Risk Manag. 2008;4(4):863-9. doi: 10.2147/vhrm.s1693.
42. Biswas S, Ghoshal PK, Mandal N. Synergistic effect of anti and pro-inflammatory cytokine genes and their promoter polymorphism with ST-elevation of myocardial infarction. Gene. 2014;544(2):145-51. doi: 10.1016/j.gene.2014.04.065.
43. Bennet AM, Prince JA, Fei GZ, Lyrenäs L, Huang Y, Wiman B, Frostegård J, Faire Ud. Interleukin-6 serum levels and genotypes influence the risk for myocardial infarction. Atherosclerosis. 2003;171(2):359-67. doi: 10.1016/j.atherosclerosis.2003.08.029.
44. Bennermo M, Nordin M, Lundman P, Boqvist S, Held C, Samnegård A, Ericsson CG, Silveira A, Hamsten A, Nastase MM, Tornvall P. Genetic and environmental influences on the plasma interleukin-6 concentration in patients with a recent myocardial infarction: a case-control study. J Interferon Cytokine Res. 2011;31(2):259-64. doi: 10.1089/jir.2010.0036.
45. Basso F, Lowe GD, Rumley A, McMahon AD, Humphries SE. Interleukin-6 -174G>C polymorphism and risk of coronary heart disease in West of Scotland coronary prevention study (WOSCOPS). Arterioscler Thromb Vasc Biol. 2002;22(4):599-604. doi: 10.1161/01.atv.0000013283.84306.1a.
46. Pramudji H, Demes CM, Dewi K, Tasmini T, Ahmad HS. Association of -174 G>C interleukin-6 gene polymorphism with interleukin-6 and c-reactive protein levels and obesity: A case-control study among people/residents of Western Indonesia. Med J Malaysia. 2019;74(5):400-404.
47. Wernstedt I, Eriksson AL, Berndtsson A, Hoffstedt J, Skrtic S, Hedner T, Hultén LM, Wiklund O, Ohlsson C, Jansson JO. A common polymorphism in the interleukin-6 gene promoter is associated with overweight. Int J Obes Relat Metab Disord. 2004;28(10):1272-9. doi: 10.1038/sj.ijo.0802763.
